# Building attention and edge message passing neural networks for bioactivity and physical–chemical property prediction

**DOI:** 10.1186/s13321-019-0407-y

**Published:** 2020-01-08

**Authors:** M. Withnall, E. Lindelöf, O. Engkvist, H. Chen

**Affiliations:** 1 Hit Discovery, Discovery Sciences, R&D, AstraZeneca, Gothenburg, Sweden; 2 Centre of Chemistry and Chemical Biology, Guangzhou Regenerative Medicine and Health-Guangdong Laboratory, 190 Kai Yuan Avenue, Science Park, Guangzhou, China

**Keywords:** Message passing neural network, Graph convolution, Virtual screening, Machine learning, Deep learning

## Abstract

Neural Message Passing for graphs is a promising and relatively recent approach for applying Machine Learning to networked data. As molecules can be described intrinsically as a molecular graph, it makes sense to apply these techniques to improve molecular property prediction in the field of cheminformatics. We introduce Attention and Edge Memory schemes to the existing message passing neural network framework, and benchmark our approaches against eight different physical–chemical and bioactivity datasets from the literature. We remove the need to introduce a priori knowledge of the task and chemical descriptor calculation by using only fundamental graph-derived properties. Our results consistently perform on-par with other state-of-the-art machine learning approaches, and set a new standard on sparse multi-task virtual screening targets. We also investigate model performance as a function of dataset preprocessing, and make some suggestions regarding hyperparameter selection.

## Introduction

QSAR (Quantitative Structure Activity Relationships) have been applied for decades in the development of relationships between physicochemical properties of chemical substances and their biological activities to obtain a reliable mathematical and statistical model for prediction of the activities of new chemical entities. The major aim of QSAR study is to reduce the number of compounds synthesized during the drug development, a notoriously long and costly process, hence the desire to improve its efficiency from a drug discovery perspective. After Hansch proposed the QSAR concept [1], engineering molecular descriptors to build accurate models for the prediction of various properties has become the standard approach to QSAR modelling. Researchers [2–6] have proposed numerous descriptors to represent molecular 2D and 3D structures, aiming to correlate these descriptors with predicted endpoints. Approaches to generating representations using the graph representation of a molecule include graph kernels [7], and perhaps most importantly in the present context, ECFP (Extended Connectivity Circular Fingerprints) [8]. Once a descriptor set has been defined, various modelling methods, including linear mapping methods like linear regression, partial least square and non-linear methods like support vector machine, random forest etc., are applied to building models. Recently, deep neural network methods have become the latest weapon in a Cheminformatician’s arsenal for doing QSAR.

Over the past decade, deep learning has become a staple in the machine learning toolbox of many fields and research areas [9, 10]. Notably in the pharmaceutical area, in recent years AI has shown incredible growth, and is being used now not just for bioactivity and physical–chemical property prediction, but also for de novo design, image analysis, and synthesis prediction, to name a few. This rapid growth is due in part to the substantial increase in available biochemical data thanks to the rise of techniques such as High Throughput Screening (HTS) and parallel synthesis, and also to the recent surge in parallel computational power that can be feasibly attained by harnessing General Purpose computing on Graphics Processing Units (GPGPU).

Efforts have also been taken to enable neural networks to do representation learning, i.e. the neural network is able to learn descriptors itself instead of relying on predefined molecular descriptors. Among these, the graph convolution network (GCN) is gaining popularity and various architectures have been proposed in data science community. The first Graph Neural Networks (GNNs) was put forward by Gori et al. in 2005 [11], presenting an architecture for learning node representations using recurrent neural networks capable of acting on directed, undirected, labelled, and cyclic graphs. This work was later expanded upon by Micheli [12] and Scarselli et al. [13] In 2013, the Graph Convolutional Network (GCN) was presented by Bruna et al. [14] using the principles of spectral graph theory. Many other forms of GNN have been presented since then, including, but not limited to, Graph Attention Networks [15], Graph Autoencoders [16–19], and Graph Spatial–Temporal Networks [20–23].

In GCNs and some other forms of GNNs, information is propagated through a graph in a manner similar to how conventional convolutional neural networks (CNNs) treat grid data (e.g. image data). However, whilst graph-based deep learning shares some connection with CNNs with respect to local connectivity of the component data, CNNs exploit the properties of regular connectivity, shift-invariance, and compositionality to achieve their noteworthy performance. In order to cope with the irregularity of graph data, alternative approaches must be designed, most notably to circumvent the issue of irregular non-Euclidean data, and to be invariant to the graph representation.

Whilst many implementations are designed for use on a single large graph, such as social networks or citation graphs, approaches designed for use on multiple smaller graphs such as graphs of small molecule are also desired for their potential use in, amongst other things, drug design. Duvenaud [24] proposed the neural fingerprint method, describing it as an analogue of ECFP, as one of the first efforts in applying graph convolution model on chemistry related problems. The notable advancement embodied in the neural fingerprint approach with regards to predecessing concepts such as graph kernels and ECFP, is that the generation of descriptors is adapted—*learned*—during training. Other molecular graph convolution methods were reported by Kearnes et al. [25] and Coley [26] as extensions to Duvenaud’s method. Recently researchers from Google [27] put forward an new NN architecture called as message passing neural networks (MPNNs) and used the MPNNs to predict quantum chemical properties. The MPNN framework contains three common steps: (1) message passing step, where, for each atom, features (atom or bond features) from its neighbours are propagated, based on the graph structure, into a so called a message vector; (2) update step, where embedded atom features are updated by the message vector; (3) aggregation step, where the atomic features in the molecule are aggregated into the molecule feature vector. These molecule feature vector can then be used in a dense layer to correlate with the endpoint property. It has been shown that the MPNN framework has a high generalizability such that several popular graph neural network algorithms [24–26, 28, 29] can be translated into the MPNN framework. Several research groups have made various extensions to the MPNN framework to augment it for work on cheminformatic problems [30].

Like GCN methods, MPNN model learns task specific molecule features from the graph structure and avoid feature engineering in the pre-processing stage. This type of method also presents an approach for the secure sharing of chemical data, i.e. it is possible to disseminate trained models for activity predictions without the risk of reverse-engineering IP-sensitive structural information [31–33].

We introduce a selection of augmentations to known MPNN architectures, which we refer to as Attention MPNN (AMPNN) and Edge Memory Neural Network (EMNN) [34], and evaluate them against published benchmark results with a range of metrics. The EMNN network shares architectural similarities to the D-MPNN model published by Yang et al. [35] that was developed concurrently to this work [36], but the D-MPNN includes additional chemical descriptor information. We applied these two types of neural network to eight datasets from the MoleculeNet [30] benchmark and analyse the performances and offer chemical justification for these results with respect to both architecture and parameter selection.

## Method

### Concepts of graphs

A graph ${\text{G}} = \left( {{\text{V}},{\text{E}}} \right)$ is a set ${\text{V}}$ of nodes and a set ${\text{E}}$ of edges, which are pairs of elements of ${\text{V}}$. If the members of E are ordered pairs, the graph is said to be directed. In the graph representation of a molecule, atoms are viewed as nodes and $\left( {v, w} \right) \in E$ indicates there is a bond between atoms $v$ and $w$. This representation is an undirected graph: we do not consider a bond to have a direction, so we do not distinguish between $\left( {v, w} \right) \,{\text{and}}\, \left( {w, v} \right)$.

In the given context, a graph comes together with a feature vector $x_{v}$ corresponding to each node $v$ and an edge feature vector $e_{vw}$ corresponding to each edge $\left( {v, w} \right)$.

### Message passing neural network

The Message Passing Neural Network [27] is a deep learning architecture designed for implementation in chemical, pharmaceutical and material science contexts. They were introduced as a framework to generalise several proposed techniques [14, 24, 25, 28, 29, 37, 38], and have demonstrated state-of-the-art results on multiple related benchmarks. For the specific MPNN implementations used for experiments in this paper, the most important predecessor is the Gated Graph Sequence Neural Network (GGNN) [28].

In simplistic terms, MPNNs operate by the following mechanism: An initial set of states is constructed, one for each node in the graph. Then, each node is allowed to exchange information, to “message”, with its neighbours. After one such step, each node state will contain an awareness of its immediate neighbourhood. Repeating the step makes each node aware of its second order neighbourhood, and so forth. After a chosen number of “messaging rounds”, all these context-aware node states are collected and converted to a summary representing the whole graph. All the transformations in the steps above are carried out with neural networks, yielding a model that can be trained with known techniques to optimise the summary representation for the task at hand.

More formally, MPNNs contain three major operations: message passing, node update, and readout. Using a message passing neural network entails iteratively updating a hidden state $h_{v} \in {\text{R}}^{\text{D}}$ of each node $v$. This is done according to the following formulas:

1 $$m_{v}^{\left( t \right)} = \mathop \sum \limits_{w \in N\left( v \right)} M_{t} \left( {h_{v}^{\left( t \right)} ,h_{w}^{\left( t \right)} ,e_{vw} } \right)$$

2 $$h_{v}^{{\left( {t + 1} \right)}} = U_{t} \left( {h_{v}^{\left( t \right)} ,m_{v}^{\left( t \right)} } \right)$$

where $M_{t}$ is the message function, $U_{t}$ is the node update function, $N\left( v \right)$ is the set of neighbours of node $v$ in graph $G$, $h_{v}^{\left( t \right)}$ is the hidden state of node $v$ at time $t$, and $m_{v}^{\left( t \right)}$ is a corresponding message vector. For each atom $v$, messages will be passed from its neighbours and aggregated as the message vector $m_{v}^{\left( t \right)}$ from its surrounding environment. Then the atom hidden state $h_{v}$ is updated by the message vector.

The formula for the readout function is shown in formula 3:

3 $$\hat{y} = R\left( {\left\{ {h_{v}^{\left( K \right)} |v \in G} \right\}} \right)$$

where $\hat{y}$ is a resulting fixed-length feature vector generated for the graph, and $R$ is a readout function that is invariant to node ordering, an important feature that allows the MPNN framework to be invariant to graph isomorphism. The graph feature vector $\hat{y}$ then is passed to a fully connected layer to give prediction. All functions $M_{t}$, $U_{t}$ and $R$ are neural networks and their weights are learned during training. While details are given in the following sections, we provide summary differences between our presented architectures in Tables 1, 2, 3 and 4.

**Table 1:** Core differences between model architectures

Model	Hidden states	Denotion of neighbourhood	Message aggregation scheme
MPNN	$h_{v}^{\left( t \right)}$	$N\left( v \right)$	$m_{v}^{\left( t \right)} = \mathop \sum \limits_{w \in N\left( v \right)} M_{t} \left( {h_{v}^{\left( t \right)} ,h_{w}^{\left( t \right)} ,e_{vw} } \right)$
AMPNN	$h_{v}^{\left( t \right)}$	$N\left( v \right)$	$m_{v}^{\left( t \right)} = A_{t} \left( {h_{v}^{\left( t \right)} ,S_{v}^{\left( t \right)} } \right)$, where $S_{v}^{\left( t \right)} = \left\{ {\left( {h_{w}^{\left( t \right)} ,e_{vw} } \right) | w \in N\left( v \right)} \right\}$
EMNN	$h_{vw}^{\left( t \right)}$	$\left\{ {\left( {k,v} \right) | k \in N\left( v \right),k \ne w} \right\}$	$m_{vw}^{\left( t \right)} = A_{t} \left( {e_{vw} ,S_{vw}^{\left( t \right)} } \right)$, where $S_{vw}^{\left( t \right)} = \left\{ {h_{kv} | k \in N\left( v \right), k \ne w} \right\}$

**Table 2:** Aggregation function special cases

Model	Hidden states	Aggregation form
MPNN	$h_{v}^{\left( t \right)}$	$M_{t} \left( {h_{v}^{\left( t \right)} ,h_{w}^{\left( t \right)} ,e_{vw} } \right) = f_{NN}^{{\left( {e_{vw} } \right)}} \left( {h_{w}^{\left( t \right)} } \right)$
AMPNN	$h_{v}^{\left( t \right)}$	$A_{t} \left( {h_{v}^{\left( t \right)} ,\left\{ {\left( {h_{w}^{\left( t \right)} ,e_{vw} } \right)} \right\}} \right) = \mathop \sum \limits_{w \in N\left( v \right)} f_{NN}^{{\left( {e_{vw} } \right)}} \left( {h_{w}^{\left( t \right)} } \right) \odot \frac{{{ \exp }\left( {g_{NN}^{{\left( {e_{vw} } \right)}} \left( {h_{w}^{\left( t \right)} } \right)} \right)}}{{\mathop \sum \nolimits_{w' \in N\left( v \right)} { \exp }\left( {g_{NN}^{{\left( {e_{vw'} } \right)}} \left( {h_{w'}^{\left( t \right)} } \right)} \right)}}$
EMNN	$h_{vw}^{\left( t \right)}$	$A_{t} \left( {e{^{\prime}}_{vw} ,S_{vw}^{\left( t \right)} } \right) = \mathop \sum \limits_{{x \in S{^{\prime}}_{vw}^{\left( t \right)} }} f_{NN} \left( x \right) \odot \frac{{{ \exp }\left( {g_{NN} \left( x \right)} \right)}}{{\mathop \sum \nolimits_{{x{^{\prime}} \in S{^{\prime}}_{vw}^{\left( t \right)} }} { \exp }\left( {g_{NN} \left( {x{^{\prime}}} \right)} \right)}}$ $S{^{\prime}}_{vw}^{\left( t \right)} = S_{vw}^{\left( t \right)} \rm{\bigcup }\left\{ {e_{vw}{^{\prime}} } \right\}$

**Table 3:** Other model architecture differences

Model	Pre-message passing	Update	Pre-readout
MPNN AMPNN	NA	$h_{v}^{{\left( {t + 1} \right)}} = {\text{GRU}}\left( {m_{v}^{\left( t \right)} ,h_{v}^{\left( t \right)} } \right)$	NA
EMNN	$e_{vw}{^{\prime}} = f_{NN}^{emb} \left( {\left( {e_{vw} ,h_{v}^{\left( 0 \right)} ,h_{w}^{\left( 0 \right)} } \right)} \right)$	$h_{vw}^{{\left( {t + 1} \right)}} = {\text{GRU}}\left( {m_{vw}^{\left( t \right)} ,h_{vw}^{\left( t \right)} } \right)$	$h_{v}^{\left( K \right)} = \mathop \sum \limits_{w \in N\left( v \right)} h_{vw}^{\left( K \right)}$

**Table 4:** Model readout function and post-readout function

Model	Readout function	Post-readout
All	$R\left( {\left\{ {\left( {h_{v}^{\left( K \right)} ,h_{v}^{\left( 0 \right)} } \right)} \right\}} \right) = \mathop \sum \limits_{v \in G} p_{NN} \left( {h_{v}^{\left( K \right)} } \right) \odot \sigma \left( {q_{NN} \left( {\left( {h_{v}^{\left( K \right)} ,h_{v}^{\left( 0 \right)} } \right)} \right)} \right)$	FFNN

### SELU message passing neural network (SELU-MPNN)

Our first architecture involved the basic MPNN framework, but with the use of the SELU activation function [39] instead of more traditional batch or layer norm functions. The SELU activation function is parameterised to converge towards a zero mean and unit variance, and removed the need to experiment with different normalisation approaches (batch, layer, tensor, etc.) explicitly. All other architectures we propose also use SELU as their activation functions. Whilst many of the graph neural network approaches presented by MolNet can be cast into the MPNN framework, we chose to use SELU-MPNN as our baseline for our implementation of the framework due to the increased convergence speed that SELU offers [40]. This affords us consistent results within our framework for a less biased comparison to more basic methods.

Apart from the different choice of activation function and hidden layers in the message function, the model we in our experiments denote SELU-MPNN shares great similarity with the original GGNN.

#### Attention message passing neural network (AMPNN)

Here we propose a further augmentation to the MPNN architecture by considering a more general form of the MPNN message summation step (Eq. 1). Using simple summation to convert an unknown cardinality set of vectors into a single vector is hypothetically an expressive bottleneck. Potential better ways to implement such aggregation functions are currently being researched [41–44]. In the current study we extend previous MPNN models for graph-level prediction by employing a straight forward aggregation function with an attention mechanism. The attention mechanism has been proposed on image recognition and language translation problems amongst others [41, 45, 46] and have achieved better performance compared with normal deep neural network algorithms. We denote our specific implementation of the extended framework an Attention Message Passing Neural Network (AMPNN). Its most important predecessor is, as for our SELU-MPNN, the GGNN [28].

As mentioned earlier, the non-weighted summation in message passing function (Eq. 1) of the original MPNN constitutes a potential limitation. In the AMPNN framework, a computationally heavier but potentially more expressive attention layer is proposed in the message passing stage to aggregate messages (Eq. 4). Equation 1 is replaced by the more general formula:

4 $$m_{v}^{\left( t \right)} = A_{t} \left( {h_{v}^{\left( t \right)} ,\left\{ {\left( {h_{w}^{\left( t \right)} ,e_{vw} } \right)|w \in N\left( v \right)} \right\}} \right)$$

where $A_{t}$ is an aggregate function invariant to the ordering of set members at step *t*. Just as for the original MPNN, the message to node $v$ is computed based on its neighbours $\left\{ {w | w \in N\left( v \right)} \right\}$, but the method of aggregation is not restricted to being a simple summation. The $A_{t}$ here chosen to be able to investigate the architecture is that of the SELU-MPNN augmented with an attention mechanism. This is mainly inspired by [41] and essentially eliminates the cardinality dimension of the set of neighbours by taking *weighted* sums. Formally, our layer is

5 $$A_{t} \left( {h_{v}^{\left( t \right)} ,\left\{ {\left( {h_{w}^{\left( t \right)} ,e_{vw} } \right)} \right\}} \right) = \mathop \sum \limits_{w \in N\left( v \right)} f_{NN}^{{\left( {e_{vw} } \right)}} \left( {h_{w}^{\left( t \right)} } \right) \odot \frac{{{ \exp }\left( {g_{NN}^{{\left( {e_{vw} } \right)}} \left( {h_{w}^{\left( t \right)} } \right)} \right)}}{{\mathop \sum \nolimits_{w' \in N\left( v \right)} { \exp }\left( {g_{NN}^{{\left( {e_{vw'} } \right)}} \left( {h_{w'}^{\left( t \right)} } \right)} \right)}}.$$

Two feed forward neural network (FFNN) $f_{NN}^{{\left( {e_{vw} } \right)}}$ and $g_{NN}^{{\left( {e_{vw} } \right)}}$ are used for each edge type $e_{vw}$ and give output vectors with the same length. The $\odot$ and the fraction bar represent Hadamard multiplication and Hadamard division, respectively. Note that because of the output dimensionality of $g_{NN}^{{\left( {e_{vw} } \right)}}$, the softmax-like operation embodied in the fraction of Eq. 5 uses a multitude of weightings rather than just one.

The $f_{NN}^{{\left( {e_{vw} } \right)}}$ network turns the hidden state of atom into an embedding vector, while the $g_{NN}^{{\left( {e_{vw} } \right)}}$ network embeds the atom hidden states into weight vectors which are turned into weight coefficients after the softmax operation. Notably, the softmax operation is done along the cardinality dimension of the set of weight vectors. Thus, the contribution of one element in the embedding vector depends on equivalent element of weight vectors in the set.

In the node update stage, similar to the GGNN, the node hidden states are updated via a gated recurrent unit, where the $m_{v}^{\left( t \right)}$ is treated as the input and the current node hidden state $h_{v}^{\left( t \right)}$ is used as the hidden state of the GRU

6 $$h_{v}^{{\left( {t + 1} \right)}} = {\text{GRU}}\left( {h_{v}^{\left( t \right)} ,m_{v}^{\left( t \right)} } \right).$$

At the initial state (t = 0), $h_{v}^{\left( 0 \right)}$ is the predefined atom feature vector. After the message passing and node updating steps are iterated for K steps, a readout function is applied to aggregate the hidden state of all the nodes in the graph into a graph level feature vector using two FFNNs. More precisely we use the GGNN readout function,

7 $$R\left( {\left\{ {\left( {h_{v}^{\left( K \right)} ,h_{v}^{\left( 0 \right)} } \right)} \right\}} \right) = \mathop \sum \limits_{v \in G} p_{NN} \left( {h_{v}^{\left( K \right)} } \right) \odot \sigma \left( {q_{NN} \left( {\left( {h_{v}^{\left( K \right)} ,h_{v}^{\left( 0 \right)} } \right)} \right)} \right)$$

where $p_{\text{NN}}$ and $q_{\text{NN}}$ are FFNNs, the $\odot$ denotes Hadamard multiplication, $\sigma$ is the sigmoid function and the (*,)* of the right hand side denotes concatenation. The generated graph feature vector is then passed into the final FFNN layer to make prediction.

#### Edge Memory Neural Network (EMNN)

**Fig. 1:**
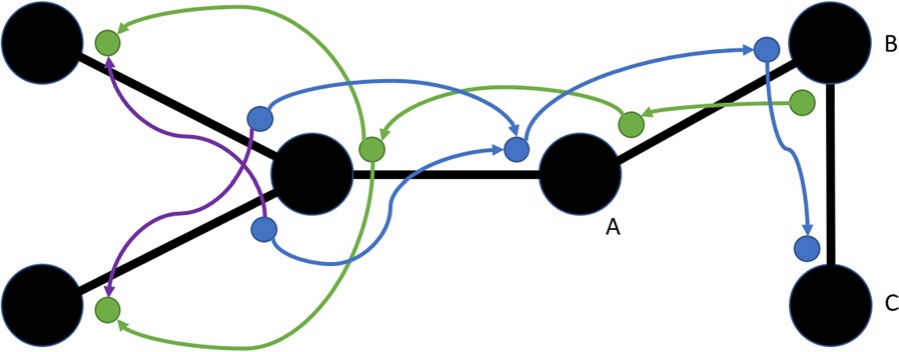
The message passing from directed neighbouring edges to another edge in EMNN. Blue and green dots represent each directed hidden state for edges. Each coloured arrow is used to represent a respective message pass within the graph—purple represents the transition from one arbitrary direction to the other when the graph branches

The message passing concept in the MPNN framework computes the message to a centre atom by aggregating information from its neighbourhood atoms in a symmetric fashion. Another MPNN-inspired model in our study has a hidden state in each directed edge (every bond has two directed edges in the *directed* graph) instead of in the nodes. In the directed graph, each bond (node–node connection) has two directed edges, thus two hidden states. The hidden state of a directed edge is updated based on hidden states of edges whose heads coincide with its tail (Fig. 1). We call this model an Edge Memory Neural Network (EMNN). In the resulting message passing step, the update of a hidden state has a corresponding direction.

This model shares underlying principles with the D-MPNN architecture proposed by Yang et al. [35] which also uses directed edges to improve MPNN performance. Their proposed model also injects additional chemical descriptor information alongside the FFNN after the message passing stage. Another notable difference between these architectures is our implementation of the afore-mentioned attention mechanism in the aggregation function. We include the D-MPNN model in our result and discussion to compare implementations and contrast the performance benefits of additional descriptor information, as has been explored in other literature [47]. We refer to their manuscript for further details on their implementation and architecture.

One hypothetical advantage compared to MPNN is explained in the following. Consider a small graph of three nodes A, B and C connected as A–B–C, as illustrated on the right-hand side of Fig. 1. If information passage from A to C is relevant to the task, two message passes are necessary with conventional MPNN. In the first pass, information is passed from A to B, as desired. However, information is also passed from C to B, so that part of B’s memory is being occupied with information that C already has. This back-and-forth passing of information happening in an MPNN hypothetically dilutes the useful information content in the hidden state of node B. When hidden states instead reside in the directed edges as per EMNN, this cannot happen. The closest thing corresponding to a hidden state in B is the hidden states in the edges $\overrightarrow {AB}$ and $\overrightarrow {CB}$. The update of $\overrightarrow {BC}$ uses information from $\overrightarrow {AB}$, but not from $\overrightarrow {CB}$.

As shown in Fig. 1, the flow of messages in each edge is directional where the message flows from a node (tail node) to another node (head node). Formally, the set of edge hidden states taken into account when updating edge $\left( {v, w} \right)$ of the directed graph $G = \left( {V, E} \right)$ is

$$S_{vw}^{\left( t \right)} = \left\{ {h_{kv} | k \in N\left( v \right), k \ne w} \right\}.$$

In the EMNN, before message passing takes place, the two node features are embedded into an edge feature by feeding a concatenation of the original edge and node feature vectors through a FFNN $f_{NN}^{emb}$,

$$e{'}_{vw} = f_{NN}^{emb} \left( {\left( {e_{vw} ,h_{v}^{\left( 0 \right)} ,h_{w}^{\left( 0 \right)} } \right)} \right)$$

At the initial state $\left( {t = 0} \right)$, $e_{vw} ,h_{v}^{\left( 0 \right)}$ are the raw bond feature vector and atom feature vector respectively and (,) refers to the concatenation operation.

The edge hidden state $h_{vw}^{\left( t \right)}$ of $\left( {v, w} \right)$ at time $t$ is updated according to Eqs. 8–10:

8 $$\left\{ {\begin{array}{*{20}l} {m_{vw}^{\left( t \right)} = A_{t} \left( {e_{vw}{^{\prime}} ,S_{vw}^{\left( t \right)} } \right)} \\ \\ {h_{vw}^{{\left( {t + 1} \right)}} = U_{t} \left( {h_{vw}^{\left( t \right)} ,m_{vw}^{\left( t \right)} } \right)} \\ \end{array} } \right..$$

Note that each directed edge has both a static edge feature $e_{vw}{^{\prime}}$ and the time-mutated edge state $h_{vw}^{\left( t \right)}$ contributing. $h_{vw}^{\left( 0 \right)}$ is instantiated as a vector of zeros. One choice of aggregation function $A_{t}$ is

9 $$A_{t}^{e} \left( {e_{vw}^{\prime } ,S_{vw}^{\left( t \right)} } \right) = \sum\limits_{{x \in S\prime_{vw}^{\left( t \right)} }} {f_{NN} } \left( x \right) \odot \frac{{{ \exp }\left( {g_{NN} \left( x \right)} \right)}}{{\sum\nolimits_{{x\prime \in S\prime_{vw}^{\left( t \right)} }} { \exp } \left( {g_{NN} \left( {x\prime } \right)} \right)}}{\mkern 1mu} \,{\text{where}}\,{\mkern 1mu} S{^{\prime}}_{vw}^{\left( t \right)} = S_{vw}^{\left( t \right)} \cup \left\{ {e_{vw}^{\prime } } \right\}$$

10 $$h_{vw}^{{\left( {t + 1} \right)}} = {\text{GRU}}\left( {h_{vw}^{\left( t \right)} ,m_{vw}^{\left( t \right)} } \right)$$

$m_{vw}^{\left( t \right)}$ is the message for edge $\left( {v,w} \right)$ at iteration $t$. $A_{t}^{e}$ is an attention based aggregation function similar to the one used in the AMPNN. $S{^{\prime}}_{vw}^{\left( t \right)}$ means all the edges involving node $v$ including the edge $\left( {v,w} \right)$ itself. Equation 10 is the update of edge $\left( {v, w} \right)$ using a GRU unit.

After $K$ message passing iterations, a node hidden state for each node is taken as the sum of the edge hidden state of edges that the node is end to,

$$h_{v}^{\left( K \right)} = \mathop \sum \limits_{w \in N\left( v \right)} h_{vw}^{\left( K \right)}$$

This is done to be able to utilize the same readout functions as seen effective for the MPNNs. The readout function for EMNN is the same as in AMPNN (Eq. 7).

#### Summary of architectural differences

All models we present are available from our git repository as abstract classes, and have been designed from the ground-up in the Pytorch [48] framework to allow modification at all points, and have been tested using CUDA libraries for GPU acceleration.

### Bayesian optimisation

Bayesian Optimisation is a method for returning the next best expected value of an N-dimensional surface by utilising all available information, in contrast to local gradient or Hessian approximation techniques. Gaussian processes are fit around datapoints as they become available, and by using suitable evaluator types, estimates of the next datapoints to be evaluated can be obtained, and a balance between surface exploration and locality optimisation can be struck. We used Expected Improvement as the acquisition function, and Local Penalisation [49] as the evaluator type in order to make batch predictions and hence explore our hyperparameter surface in parallel. The hyperparameters used in the NN were tuned using the Bayesian optimization package GPyOpt [50].

The hyperparameters searched in Bayesian optimization and their constrained ranges are listed in Table 5. Due to architectural differences and an increased number of parameters, the optimisation range for the EMNN was slightly tightened.

**Table 5:** A list of hyperparameters optimised for each architecture type, and the domains over which they were optimised

Hyperparameter	SELU-MPNN	AMPNN	EMNN
Learn-rate	{$10^{ - 6} - 10^{ - 4}$}	{$10^{ - 6} - 10^{ - 4}$}	{$10^{ - 6} - 10^{ - 4}$}
Message-size	[10,16,25,40]	[10,16,25,40]	NA
Message-passes	[1–10]	[1–10]	[1–8]
Msg-hidden-dim	[50,85,150]	[50,85,150]	[50,85,150]
Gather-width	[30,45,70,100]	[30,45,70,100]	[30,45,70,100]
Gather-emb-hidden-dim	[15,26,45,80]	[15,26,45,80]	[15, 26, 45]
Gather-att-hidden-dim	[15,26,45,80]	[15,26,45,80]	[15, 26, 45]
Out-hidden-dim	[360,450,560]	[360,450,560]	[360,450,560]
Out-dropout-p	{0.0–0.1}	{0.0–0.1}	{0.0–0.1}
Out-layer-shrinkage	{0.2–0.6}	{0.2–0.6}	{0.2–0.6}
Att-hidden-dim	NA	[50,85,150]	[50,85,150]
Edge-emb-hidden-dim	NA	NA	[60,105,180]
Edge-embedding-size	NA	NA	[30,50,80]

Square brackets indicate discrete domains

*NA* not applicable

### Datasets

We used a selection of 8 datasets presented in the MoleculeNet (MolNet) [30] benchmarking paper to evaluate the networks. Datasets (shown in Table 6) were split according to the methods described in the MolNet paper. Datasets were split either randomly, or by Bemis-Murcko scaffold [51]. In the case of randomly split sets, three sets were produced, split by fixed random seeds. Each dataset was split into train/test/validation sets in the ratio 80/10/10 as per the MolNet procedure. Optimal hyperparameters were determined based on their performance on the validation set of the primary split. Once optimal hyperparameters were selected three models were trained, one for each split, and the test scores for the best validation set epoch were averaged and the standard deviation calculated. In the case of scaffold splitting, test runs were still performed three times, and variation in the runs is the result of randomly initiated weights and biases. Each task in each dataset was normalised prior to training, and the results were transformed back after being passed through the model. Normalisation was done the same way as MolNet, with the notable exception of QM8.^1^ The node features generated from the datasets were: Atom Type, Atom Degree, Implicit Valence, Formal Charge, Number of Radical Electrons, Hybridization (SP, SP2, SP3, SP3D, SP3D2), Aromaticity, and Total Number of Hydrogens. These features were generated as per the MolNet Deepchem functions. For edge features, the bond types were limited to single bonds, double bonds, triple bonds and aromatic bonds.

**Table 6:** The selection of datasets on which models were trained, and details pertaining to these sets

Dataset	Tasks	Type	Compounds	Split	Metric
MUV	17	Classification	93,127	Random	PRC-AUC
HIV	1	Classification	41,913	Scaffold	ROC-AUC
BBBP	1	Classification	2053	Scaffold	ROC-AUC
Tox21	12	Classification	8014	Random	ROC-AUC
SIDER	27	Classification	1427	Random	ROC-AUC
QM8	12	Regression	21,786	Random	MAE
ESOL	1	Regression	1128	Random	RMSE
LIPO	1	Regression	4200	Random	RMSE

The QM8 dataset [52] contains electronic spectra calculated from coupled-cluster (CC2) and TD-DFT data on synthetically feasible small organic molecules. The ESOL [53] dataset comprises aqueous solubility values for small molecules, “medium” pesticide molecules, and large proprietary compounds from *in*-*house* Syngenta measurements. The LIPO dataset comprises lipophilicity data. The MUV dataset [54] contains PubChem bioactivity data specially selected and arranged by refined nearest-neighbour analysis for benchmarking virtual screening approaches. The HIV dataset [55] comprises classification data for compound anti-HIV activity. The BBBP dataset [56] contains data regarding compound ability to penetrate the blood–brain barrier. The Tox21 dataset [57] was released as a data analysis challenge to predict compound toxicity against 12 biochemical pathways. The SIDER set [58] is a collection of drugs and corresponding potential adverse reactions grouped following MedDRA classifications [59] according to previous usage [60].

### Preprocessing

**Fig. 2:**
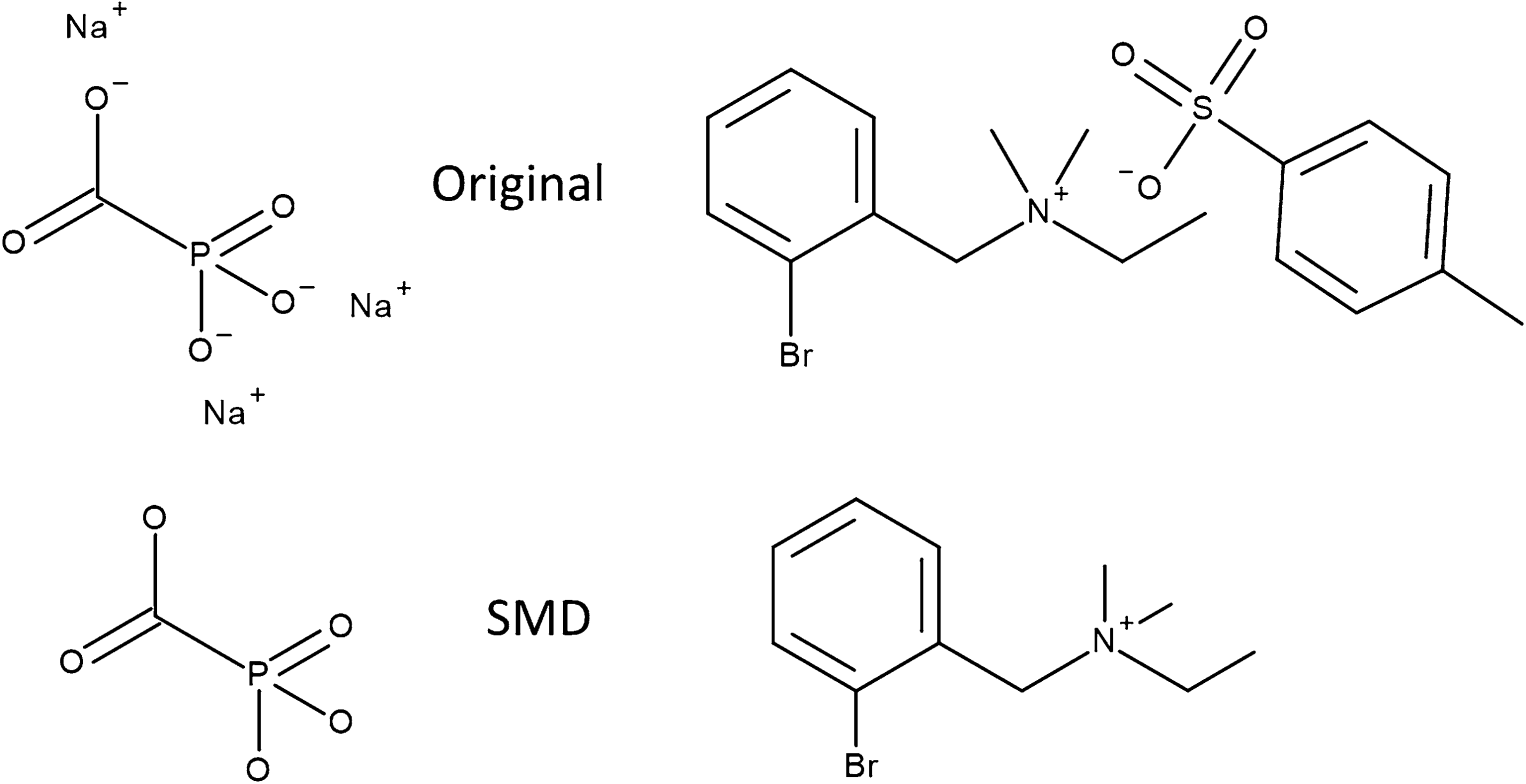
Examples of ionic complexes found in the datasets, and their charge-parent standardized counterparts, as used in the SMD datasets

Datasets were used both directly as provided from the MolNet repository without any preprocessing, and with some preprocessing procedure. Dataset preprocessing constituted transformation of the given SMILES string to that of the standardised charge-parent molecule, and reintroduction of ‘missing value’ labels where appropriate in multitask sets, which we refer to as SMD (Standardised Missing Data) preprocessing (Fig. 2). Charge-parent fragmentation was performed using the MolVS standardizer [61], which returned the uncharged version of the largest organic covalent unit in the molecule or complex. In the original datasets, these values were imputed as inactive as per previous literature. The reintroduction of ‘missing value’ labels allows the use of a masking loss function that operates over the set [Active, Inactive, Missing] and does not include missing data in the loss calculation. This prevents backpropagation of molecule-target information in multitask datasets when it is not available.

## Results

We present our results as a comparison against the MoleculeNet paper [30], showing test set performances and relative test set errors to the best reported graph-based MoleculeNet architecture, as well as other classical machine learning models. We show our architectures (SELU-MPNN, AMPNN and EMNN models) for both the unaltered and for the SMD preprocessed data, compared against the literature values for the original datasets to allow for fair benchmarking comparison for both the methods and for the preprocessing approaches. Complete tables are available in Additional file 1, alongside model performance information and statistical tests. The results from the literature for other machine learning methods were also reported to have hyperparameters optimised by the authors, using Bayesian Optimisation where applicable, so should present a fair comparison. Some techniques are missing for some larger datasets; this is because they were not reported in the original publications, presumably due to computational limits. Our runs were performed only for the models we present, and these are compared against values taken from literature benchmark studies for other models.

**Fig. 3:**
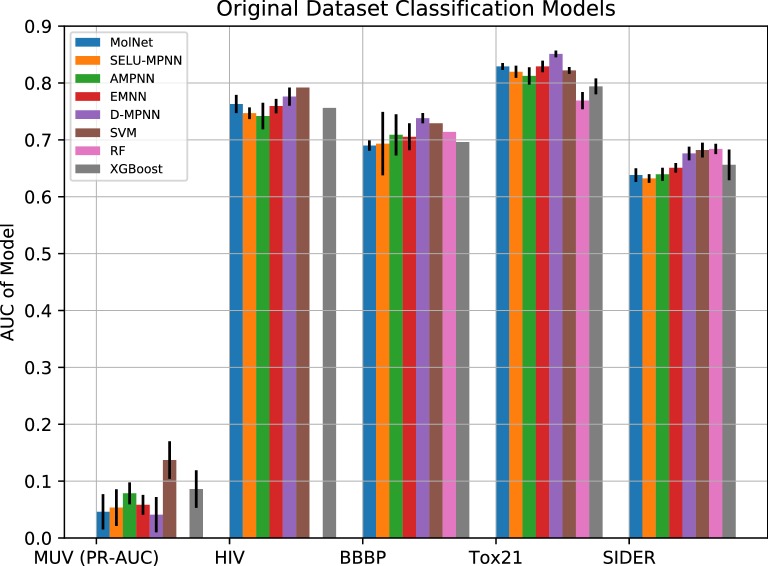
Predictive performances of machine-learning approaches relative to the best MolNet graph model. With the exception of MUV, the metric used is ROC-AUC. The higher the y-axis is, the better the model performs

Performance in terms of AUC in classification on the original dataset was on par with state of the art for the majority of models, with the exception of the MUV set (Fig. 3), where a modest increase in performance was observed relative to MolNet. However, this increase was not significant compared to Support-Vector Machines, which had the highest performance by a large margin. The AMPNN architecture was the best of our presented approaches, with the third highest overall performance on the MUV dataset. The D-MPNN showed a mild performance increase over our architectures for sets other than MUV.

**Fig. 4:**
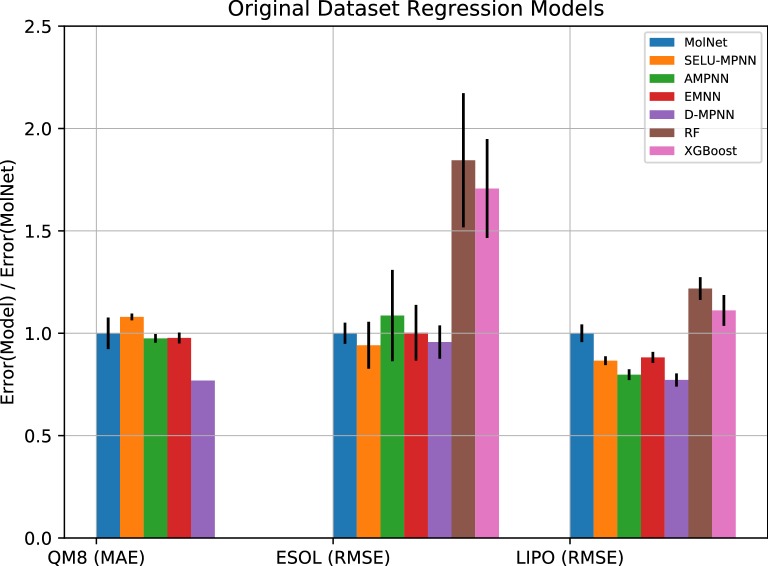
Regression errors of machine-learning approaches relative to the best MolNet graph model. Metrics are specified for each dataset. The lower the y-axis is, the better the model performs

In terms of regression on the original datasets (Fig. 4), the AMPNN was also one of the best performing architectures we present, achieving the lowest error with smallest variance on two of the three sets, covering single and multi-task problems. Performance on the QM8 and ESOL datasets over our three presented architectures was more-or-less on par with MolNet, performing better than Random Forest and XGBoost models, and being beaten by the D-MPNN consistently. However, on the lipophilicity set, all our presented architectures achieved a lower error than all other presented approaches excepting the D-MPNN, which was rivalled by the AMPNN implementation. The Random Forest and XGBoost results are to be expected, as these approaches are much more suited to classification than regression.

**Fig. 5:**
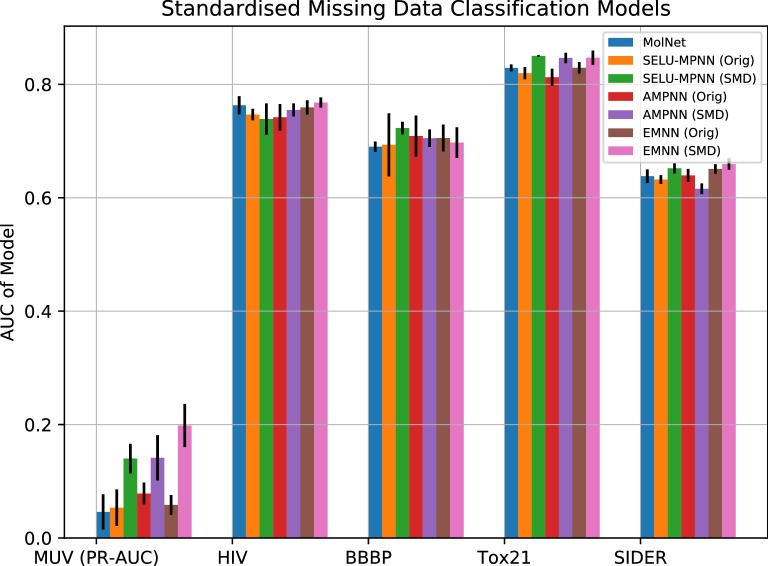
Predictive performances of our machine-learning approaches on the SMD sets relative to MolNet and the respective original models. With the exception of MUV, the metric used is ROC-AUC. The higher the y-axis is, the better the model performs

Performance in classification on the SMD preprocessed dataset was also on par with state of the art for the majority of models, again with the exception of the MUV set (Fig. 5). Little change was observed between the preprocessing techniques for the rest of the datasets, with minor improvement observed in the Tox21 models, a couple of the SIDER and HIV models, and one BBBP model. However, the MUV performance was considerably increased, with two of our architectures (SELU-MPNN and AMPNN) performing as well as SVM model, at three times the predictive power of the presented MolNet architecture. The EMNN network was the best performing architecture, beating SVM models and presenting a predictive power on average over four times higher than MoleculeNet original performance, with only a slightly higher variance.

**Fig. 6:**
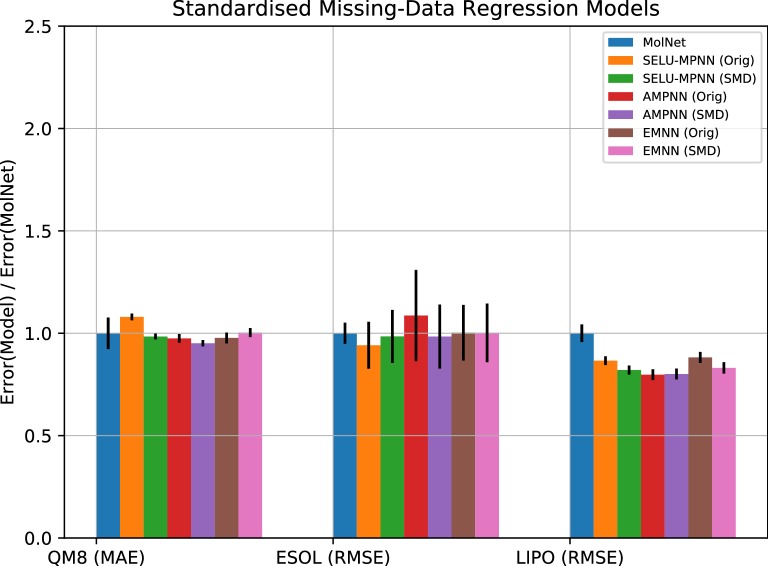
Regression errors of our machine-learning approaches for the SMD sets relative to MolNet and the respective original models. Metrics are specified for each dataset. The lower the y-axis is, the better the model performs

Regression on the SMD datasets (Fig. 6) also showed a little improvement overall versus the original datasets. The AMPNN was again one of the best performing architectures we present, achieving the lowest error with the smallest variance of the SMD models on the same two of the three sets as before, and showing a marked improvement on the ESOL dataset with this preprocessing approach. The lipophilicity set also showed lower overall error with these approaches, though the improvement is minor compared to the improved performance in classification.

Overall, we have demonstrated increased predictive power for some of our architectures dependent on task modelled. We have also demonstrated an improved dataset preprocessing technique that can increase the modelling capabilities of our networks under certain circumstances.

## Discussion

### Datasets

#### Classification

**Fig. 7:**
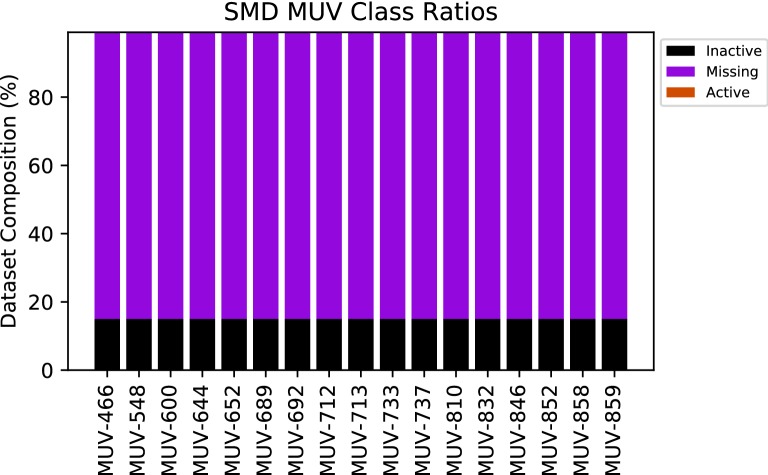
Ratio of actives, inactives, and missing data for each task in the MUV dataset. Actives represent such a small proportion that they are not visible in this diagram

The reintroduction of missing data labels is likely the cause of the increased MUV performance over other methods. As shown in Table 7 and Fig. 7, approximately 84% of the data points in the MUV multitask set are unlabelled. In the original datasets, these points are imputed as inactives, which may introduce a large erroneous class imbalance to the dataset and affect performance.

**Table 7:** Number of Actives, inactives, and missing datapoints in the classification sets used in the study

Classification set	Number of actives	Missing datapoints	Number of inactives
MUV	398	1,066,216	199,359
HIV	1232	0	31,669
BBBP	1341	0	290
Tox21	4617	12,821	57,730
SIDER	17,440	0	13,367

**Table 8:** Task Information for the MUV dataset

Task label	Target	Mode of interaction	Target class	Assay type
MUV-466	S1P1 rec.	Agonists	GPCR	Reporter Gene
MUV-548	PKA	Inhibitors	Kinase	Enzyme
MUV-600	SF1	Inhibitors	Nuclear Receptor	Reporter Gene
MUV-644	Rho-Kinase2	Inhibitors	Kinase	Enzyme
MUV-652	HIV RT-RNase	Inhibitors	RNase	Enzyme
MUV-689	Eph rec. A4	Inhibitors	Rec. Tyr. Kinase	Enzyme
MUV-692	SF1	Agonists	Nuclear Receptor	Reporter Gene
MUV-712	HSP 90	Inhibitors	Chaperone	Enzyme
MUV-713	ER-a-coact. bind.	Inhibitors	PPIc	Enzyme
MUV-733	ER-β-coact. bind.	Inhibitors	PPIc	Enzyme
MUV-737	ER-a-coact. bind.	Potentiators	PPIc	Enzyme
MUV-810	FAK	Inhibitors	Kinase	Enzyme
MUV-832	Cathepsin G	Inhibitors	Protease	Enzyme
MUV-846	FXIa	Inhibitors	Protease	Enzyme
MUV-852	FXIIa	Inhibitors	Protease	Enzyme
MUV-858	D1 rec.	Allosteric modulators	GPCR	Reporter Gene
MUV-859	M1 rec.	Allosteric inhibitors	GPCR	Reporter Gene

When treating missing data as inactive in the original datasets, actives represent only 0.03% of the dataset, whereas ignoring missing data as with SMD sets the actives represent approximately 0.2% of the dataset, nearly an order of magnitude more. Heavily unbalanced datasets are notoriously tricky to train models on, and a reduction of this bias may explain the performance improvements of SMD processed data over the original MUV dataset.

**Fig. 8:**
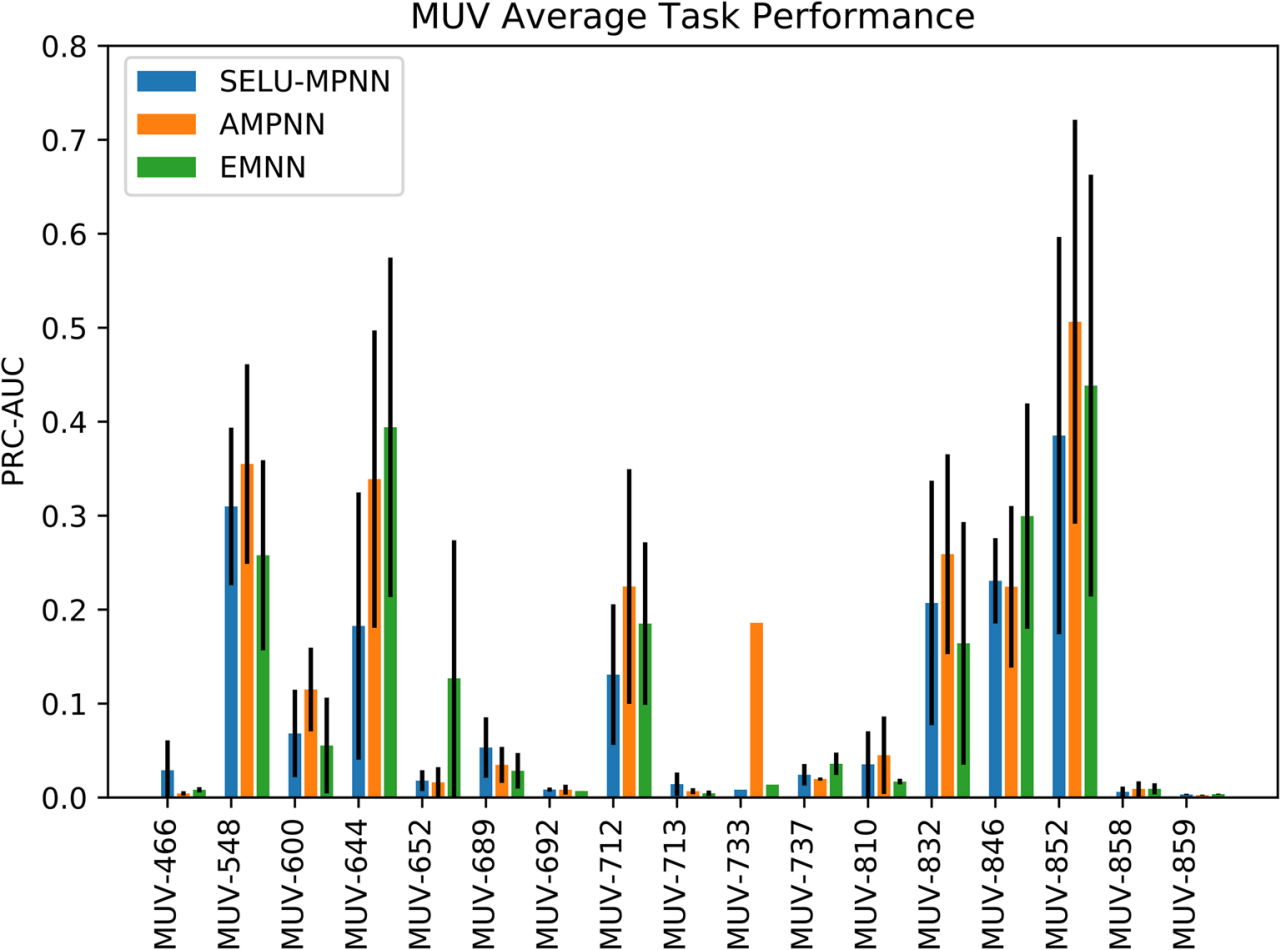
Per-task results for the SMD MUV test set. Translations between task label and target information are available in Table 8

As the SMD MUV dataset greatly outperformed other deep-learning approaches, we present a deeper analysis on this set. Per-task results (Fig. 8) ranged between minimal learned knowledge and well-learned knowledge when averaged across the three runs, and were on the whole very consistent between architectures. Tasks 548 and 644, and tasks 832, 846 and 852 are of particular note: These correspond to Kinase Inhibitors and Protease Inhibitors respectively, and are our highest-performing tasks with the exception of task 712.

**Fig. 9:**
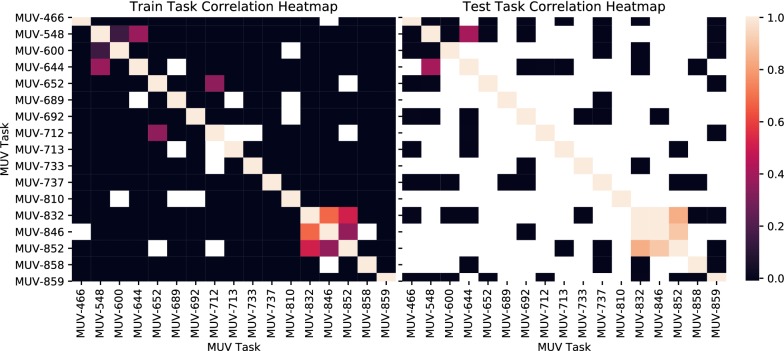
Correlation heatmaps between tasks for the training and test sets. These have been averaged across all splits. White indicates no data available for correlation (at least one missing datapoint for all pairs)

An analysis of these tasks gave a greater insight into one reason for the performance boost. As shown in Fig. 9, these tasks had a much greater activity correlation than others, i.e. ligands observed to be active or inactive for these tasks were likely to share similar activity with the others. This allows the network to much more effectively pick up on common structural features and learn them as reported in other studies [62, 63]. However, in the case where missing data is imputed as inactive, these correlations become more difficult to learn, as negative counterexamples examples are artificially introduced. Other tasks, such as the PPIc or GPCR tasks, are more challenging to learn; by the nature of the target, the structural diversity of the actives compounded with the sparsity of the data, the class imbalances and the lack of transfer learning examples, results in very low performance.

The other tasks display generally poor activity, or occasional performance peaks. Due to the extremely limited number of active compounds per task in the test-set, these performance peaks are expected to be sporadic and not true signal. Indeed, for task MUV-733, there were no active compounds in the test set for two of the three splits^2^ as split by MolNet procedure. As a method for improving performance, for future work we suggest encoding structural features of the target alongside the ligand may be one approach that could be used when correlated target information is not available.

The imputation of missing data as inactives in smaller sets with fewer missing labels has a much smaller impact. Tox21, with only approximately 17% missing data, has a barely perceptible change in active/inactive ratios when missing data is ignored—changing from 6.1% active to 7.4% (Additional file 1). The performance increase here is therefore more likely to be due to false imputation of inactives in the dataset disrupting the learning process and making learning molecular features harder, than it is to be from a confusion of transfer learning examples.

The SIDER (no missing labels) performance demonstrates our algorithms are remarkably resilient to multiple unbalanced sets in a multitask setting, performing on par with most other contemporary machine learning algorithms (Additional file 1). They maintain an advantage even against algorithms that must be trained as multiple single-task models instead of a singular multitask algorithm. The performance increase between the Original and SMD datasets was found to be negligible.

The networks perform on-par with other approaches for single-task classification—the HIV and BBBP classification sets. During the dataset analysis we observed that some compounds exist in counterionic forms in some datasets, which may not be optimal for ADMETox modelling: the charge-parent aspect of the SMD preprocessing was introduced to convert molecules to more pharmacologically-relevant forms as they may exist in the body. This was naïvely done by removing complexes from the datasets, notably ionic complexes such as those shown in Fig. 2, under the assumption that the largest fragment contributes the effect, and to ensure the consistency of charge representation. Further, there was an initial concern that, as ionic bonds are not modelled in the models’ edge types, information would not be able to propagate between the disjoint components of the complex, and smaller components such as the sodium ions would act as artefacts in the graph and introduce noise. However, the lack of performance difference between the two suggests that the readout function bridged these gaps successfully, and the network can be robust against multiple fragments. As well as HIV and BBBP, this is supported by the negligible performance difference between the SIDER models of the two sets.

#### Regression

**Fig. 10:**
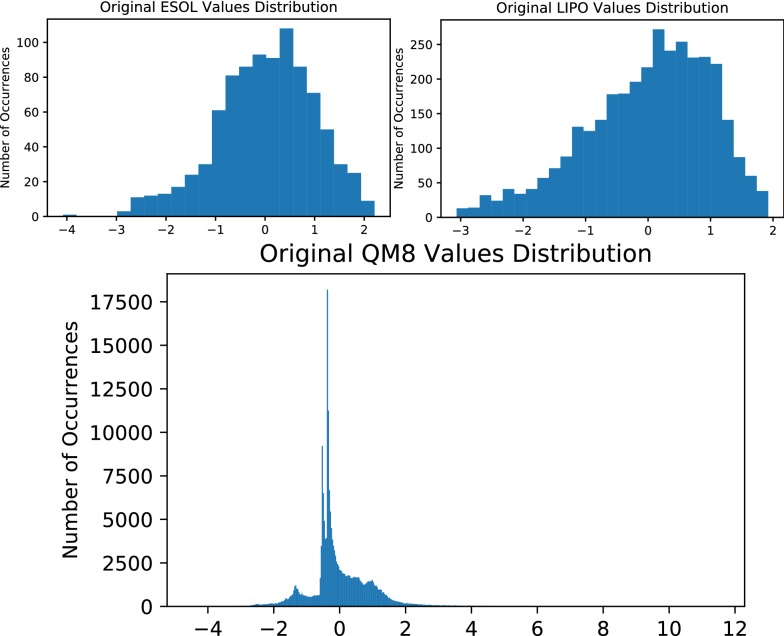
Distribution of property values from the ESOL, LIPO and QM8 regression datasets after normalisation by mean and standard deviation

The models performed in general on-par with existing models in regression modelling, with a significant reduction in error when working on the LIPO dataset. The models seem robust against various distributions of values, with ESOL and LIPO datasets resembling skewed normal distributions and QM8 resembling a much more atypical distribution, with most values centred in a singular narrow range close to zero (Fig. 10).

It is not known whether improvement can be further gained in some of these modelled tasks. The ESOL solubility models, for example, are close to the estimated experimental error of the original data. The estimated experimental error of drug-like compound solubility is usually cited as an RMSE around 0.6 logS units [64]. Simpler molecules nevertheless can be modelled with a much lower error around 0.3–0.4 log units [65]—this same study further suggests that the limit of ca. 0.6 log units for drug-like compounds may not be due to experimental or data curation issues, but a limit of QSPR modelling as applied to these databases. The creation of large datasets suitable for training complex models with lower experimental error is a nontrivial task, as solubility is a difficult property to measure correctly in a high throughput scenario: The ‘gold-standard’ measure for solubility—the shake-flask method, is a comparatively costly and time-consuming approach.

In contrast to the estimation of error for experimental physical chemical properties, other datasets can be difficult to give a lower bound of error, for example the QM8 dataset. DFT is in theory exact, however in practice a small but important energy component must be approximated. Though modern approximations provide useful accuracy for practical purposes, errors are not strictly variational, so systematic improvement is problematic. Compounding this, practical implementations introduce other errors (from e.g. choice of basis set, grid resolution), and as such quantifying the limit of how well neural networks can model these properties is difficult.

### Hyperparameters

**Fig. 11:**
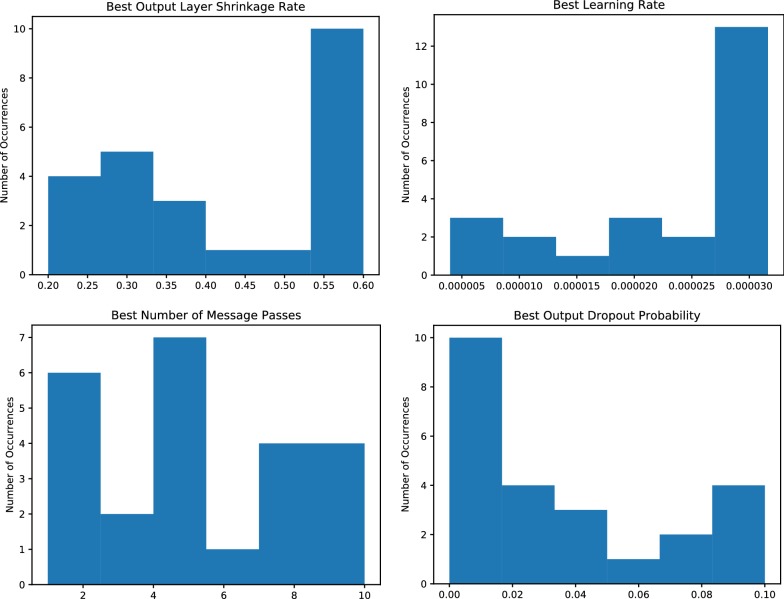
Aggregate distributions of hyperparameters observed over all tasks and architectures on the SMD datasets after optimisation

Due to the extensive hyperparameter optimisation that was performed during the training process, we analysed the distributions of hyperparameters to see if there were any tendencies towards optimal configurations for future work. Of the optimised hyperparameters (Table 5) we found that the shrinkage rate of the output fully-connected layer, the learning rate, the number of message passing iterations, and the output layer dropout rate were of note (Fig. 11). Other hyperparameters did not display any notable trends.

We found that generally a higher output layer shrinkage rate and a higher learning rate was more optimal for network performance. The learning rate was often hitting the maximum allowed value of the specified optimisation domain, which may indicate that performance could be further improved if this limit was expanded, pushing the distribution towards a more uniform coverage.

Conversely, dropout was observed to be generally lower in optimal hyperparameters across model training. Whilst this may generally be undesirable as it can lead to model overfitting, the evaluation of the model in a train/test/validation splitting approach should penalise any tendencies to overfit. This would imply that other aspects of the MPNN architecture act as feature regularisation and prevent this, though this cannot be stated conclusively. Figures supplied in the ESI suggest that no notable overfitting was observed during training, which may give the approach inherent advantages over machine learning methods that are traditionally more prone to overfitting. The number of message passes did not show any clear trend, and can be assumed to be heavily dependent on task and other hyperparameters. Some tasks such as ESOL and Tox21 however showed a small bias towards fewer message passing iterations, which makes sense as features such as hydrogen bond donors/acceptors, toxicophores etc. can be very localised and large contributing factors to these properties.

## Conclusion

We have introduced two augmentations to the MPNN framework that have shown performance on-par or greater than existing benchmarking models. One is the Attention MPNN, and the other the Edge Memory NN, both of which performed competitively with state of the art machine learning techniques of both traditional and deep learning varieties. The introduction of the attention scheme to our baseline MPNN framework added minimal model overhead, and offers no disadvantages for its use compared to the baseline model, in situations where it is effective. The EMNN had computational cost disadvantages, however, its use may be justified in situations where it offers significant performance increases: We demonstrate that our algorithms can outperform state-of-the-art models in virtual screening settings, notably demonstrated on sparse multi-task datasets, even without the inclusion of target structural information. Further, the inclusion of an attention mechanism may aid in model interpretability, as explored in other literature [66]. We were fairly consistently outperformed by the analogous D-MPNN architecture on other tasks, however we noted generally comparable performance without the inclusion of additional chemical descriptor information, using only low-level chemical graph data. We have analysed different approaches to multitask modelling and dataset preprocessing that have demonstrated increased performance under specific conditions, most notably presenting that the graceful handling of missing data can contribute significantly to model performance in highly sparse datasets. Further, we have performed an extensive hyperparameter optimisation over many model parameters and provided a summary analysis of some more common hyperparameters, indicating potential starting values for future work.

## Supplementary information

**Additional file 1.** Additional tables and figures.

## Data Availability

The code we used in this paper is published and available at https://github.com/edvardlindelof/graph-neural-networks-for-drug-discovery.

## Footnotes

1 In other regression datasets, the dataset was normalised, and then split into train/test/validation splits, whereas QM8 was split and then each split normalised. We chose to normalise QM8 in the same manner as the other regression sets, splitting after normalising over the whole set.

2 In future work, to mitigate these issues in highly sparse, highly unbalanced datasets, we encourage the use of alternative splitting approaches such as stratified sampling. Alternatively, if random sampling is preferring, repeat selection of seeds until at least one active is available for each task is recommended.
