# Supplementary material for: Building attention and edge message passing neural networks for bioactivity and physical–chemical property prediction
Source: J Cheminform. 2020 Jan 8;12:1. doi: 10.1186/s13321-019-0407-y (PMC6951016; doi:10.1186/s13321-019-0407-y)
Supplement: Supplementary file 1 — Additional file 1. Additional tables and figures. [file 13321_2019_407_MOESM1_ESM.docx]

# Additional Information

## Complete Model Prediction Values

Table S1 – Complete Results for All Regression Sets (Lower is Better)

| Dataset | Model | Training Score | Validation Score | Test Score |
| --- | --- | --- | --- | --- |
| QM8  (MAE) | SELU-MPNN | 0.0150±0.0002 | 0.0157±0.0003 | 0.0154±0.0002 |
|  | AMPNN | 0.0133±0.0001 | 0.0141±0.0003 | 0.0139±0.0003 |
|  | EMNN | 0.0136±0.0001 | 0.0142±0.0003 | 0.0140±0.0004 |
| QM8-SMD  (MAE) | SELU-MPNN | 0.0133±0.0001 | 0.0142±0.0002 | 0.0141±0.0002 |
|  | AMPNN | 0.0127±0.0003 | 0.0137±0.0001 | 0.0136±0.0002 |
|  | EMNN | 0.0138±0.0002 | 0.0144±0.0001 | 0.0143±0.0003 |
| ESOL  (RMSE) | SELU-MPNN | 0.394±0.013 | 0.541±0.047 | 0.546±0.066 |
|  | AMPNN | 0.450±0.025 | 0.563±0.033 | 0.630±0.129 |
|  | EMNN | 0.402±0.033 | 0.538±0.005 | 0.581±0.079 |
| ESOL-SMD  (RMSE) | SELU-MPNN | 0.328±0.023 | 0.509±0.025 | 0.571±0.075 |
|  | AMPNN | 0.429±0.081 | 0.583±0.022 | 0.571±0.091 |
|  | EMNN | 0.371±0.084 | 0.537±0.010 | 0.581±0.083 |
| LIPO  (RMSE) | SELU-MPNN | 0.373±0.020 | 0.617±0.057 | 0.623±0.016 |
|  | AMPNN | 0.346±0.027 | 0.595±0.039 | 0.573±0.019 |
|  | EMNN | 0.511±0.024 | 0.699±0.034 | 0.634±0.020 |
| LIPO-SMD  (RMSE) | SELU-MPNN | 0.359±0.008 | 0.637±0.033 | 0.590±0.016 |
|  | AMPNN | 0.367±0.013 | 0.604±0.056 | 0.576±0.019 |
|  | EMNN | 0.403±0.013 | 0.613±0.041 | 0.597±0.020 |

Table S2 – Complete Results for All Classification Sets (Higher is Better)

| Dataset | Model | Training Score | Validation Score | Test Score |
| --- | --- | --- | --- | --- |
| MUV  (PRC-AUC) | SELU-MPNN | 0.311±0.197 | 0.055±0.030 | 0.053±0.032 |
|  | AMPNN | 0.296±0.152 | 0.059±0.019 | 0.079±0.019 |
|  | EMNN | 0.304±0.234 | 0.051±0.020 | 0.058±0.017 |
| MUV-SMD  (PRC-AUC) | SELU-MPNN | 0.405±0.154 | 0.131±0.032 | 0.140±0.026 |
|  | AMPNN | 0.770±0.195 | 0.136±0.040 | 0.141±0.040 |
|  | EMNN | 0.691±0.051 | 0.147±0.020 | 0.198±0.038 |
| HIV  (ROC-AUC) | SELU-MPNN | 0.850±0.026 | 0.816±0.002 | 0.747±0.010 |
|  | AMPNN | 0.868±0.018 | 0.801±0.016 | 0.742±0.023 |
|  | EMNN | 0.841±0.013 | 0.808±0.014 | 0.759±0.013 |
| HIV-SMD  (ROC-AUC) | SELU-MPNN | 0.847±0.058 | 0.755±0.033 | 0.739±0.028 |
|  | AMPNN | 0.879±0.006 | 0.802±0.009 | 0.755±0.012 |
|  | EMNN | 0.867±0.031 | 0.820±0.023 | 0.768±0.009 |
| BBBP  (ROC-AUC) | SELU-MPNN | 0.942±0.009 | 0.956±0.011 | 0.693±0.056 |
|  | AMPNN | 0.919±0.038 | 0.960±0.008 | 0.709±0.036 |
|  | EMNN | 0.926±0.018 | 0.962±0.005 | 0.705±0.024 |
| BBBP-SMD  (ROC-AUC) | SELU-MPNN | 0.919±0.003 | 0.966±0.001 | 0.723±0.011 |
|  | AMPNN | 0.937±0.010 | 0.963±0.006 | 0.705±0.015 |
|  | EMNN | 0.910±0.012 | 0.959±0.005 | 0.697±0.027 |
| Tox21  (ROC-AUC) | SELU-MPNN | 0.894±0.011 | 0.832±0.009 | 0.820±0.011 |
|  | AMPNN | 0.875±0.019 | 0.832±0.012 | 0.812±0.015 |
|  | EMNN | 0.889±0.011 | 0.843±0.006 | 0.829±0.010 |
| Tox21-SMD  (ROC-AUC) | SELU-MPNN | 0.929±0.012 | 0.863±0.004 | 0.850±0.001 |
|  | AMPNN | 0.918±0.006 | 0.862±0.005 | 0.846±0.009 |
|  | EMNN | 0.895±0.002 | 0.861±0.011 | 0.847±0.013 |
| SIDER  (ROC-AUC) | SELU-MPNN | 0.681±0.008 | 0.621±0.015 | 0.632±0.008 |
|  | AMPNN | 0.742±0.028 | 0.638±0.022 | 0.639±0.011 |
|  | EMNN | 0.783±0.027 | 0.641±0.019 | 0.651±0.008 |
| SIDER-SMD  (ROC-AUC) | SELU-MPNN | 0.791±0.057 | 0.645±0.011 | 0.652±0.009 |
|  | AMPNN | 0.668±0.047 | 0.615±0.020 | 0.616±0.009 |
|  | EMNN | 0.772±0.017 | 0.646±0.019 | 0.659±0.010 |

Table S3 - Statistics associated with regression datasets. The two sample Welch's t-test was used to calculate associated p-values.

| **Dataset** | **Method** | **P(Original dataset model not better than MolNet)** | **P(SMD model not better than respective Original model)** |
| --- | --- | --- | --- |
| **MUV** | SELU-MPNN | 0.396 | 0.018 |
|  | AMPNN | 0.110 | 0.046 |
|  | EMNN | 0.296 | 0.005 |
| **HIV** | SELU-MPNN | 0.880 | 0.665 |
|  | AMPNN | 0.856 | 0.225 |
|  | EMNN | 0.622 | 0.193 |
| **BBBP** | SELU-MPNN | 0.466 | 0.214 |
|  | AMPNN | 0.221 | 0.564 |
|  | EMNN | 0.190 | 0.638 |
| **Tox21** | SELU-MPNN | 0.853 | 0.008 |
|  | AMPNN | 0.915 | 0.022 |
|  | EMNN | 0.500 | 0.075 |
| **SIDER** | SELU-MPNN | 0.741 | 0.031 |
|  | AMPNN | 0.462 | 0.963 |
|  | EMNN | 0.111 | 0.183 |

Table S4 - Statistics associated with classification datasets. The two sample Welch's t-test was used to calculate associated p-values.

| **Dataset** | **Method** | **P(Original dataset model not better than MolNet)** | **P(SMD model not better than respective Original model)** |
| --- | --- | --- | --- |
| **QM8** | SELU-MPNN | 0.906 | 0.003 |
|  | AMPNN | 0.293 | 0.130 |
|  | EMNN | 0.343 | 0.815 |
| **ESOL** | SELU-MPNN | 0.239 | 0.653 |
|  | AMPNN | 0.720 | 0.282 |
|  | EMNN | 0.508 | 0.500 |
| **LIPO** | SELU-MPNN | 0.009 | 0.041 |
|  | AMPNN | 0.003 | 0.571 |
|  | EMNN | 0.014 | 0.052 |

## Relative Model Computational Costs

The majority of calculations were performed on a variety of compute nodes, comprising Broadwell and Skylake CPUs and NVIDIA K80 and V100 GPUs. In order to present comparable benchmarking results, we reran a selection of models on a workstation, with a Quadro P5000 GPU, 32GB of RAM, and an Intel Xeon E5-1620 v3 @ 3.5Ghz. All runs used identical hyperparameters, and were run for 1000 epochs. As the MPNN model complexity varies with respect to input graph size and hyperparameters, we present the G-RAM usage during training, as measured using nvidia-smi --query-compute-apps.

| Dataset | Number of Molecules | Average Atoms Per Molecule | Architecture | Total Time (hh:mm:ss) | Time per Epoch (mm:ss.ms) | Time per Epoch per Mol (ms) |
| --- | --- | --- | --- | --- | --- | --- |
| BBBP (SMD) | 2053 | 23.9 | SELU-MPNN | 01:21:05 | 00:04.865 | 2.370 |
|  |  |  | AMPNN | 01:27:31 | 00:05.251 | 2.558 |
|  |  |  | EMNN | 09:34:06 | 00:34.446 | 16.778 |
| SIDER (SMD) | 1427 | 32.3 | SELU-MPNN | 01:45:58 | 00:06.358 | 4.456 |
|  |  |  | AMPNN | 01:52:23 | 00:06.743 | 4.725 |
|  |  |  | EMNN | 14:25:10 | 00:51.910 | 36.377 |
| Tox21 (SMD) | 8014 | 19.2 | SELU-MPNN | 03:21:01 | 00:12.061 | 1.505 |
|  |  |  | AMPNN | 03:34:41 | 00:12.881 | 1.607 |
|  |  |  | EMNN | 24:31:14 | 01:28.274 | 11.015 |

| Dataset | Average Atoms Per Molecule | Number of Tasks | Architecture | G-RAM Min | G-RAM Max | G-RAM Avg (Mode) |
| --- | --- | --- | --- | --- | --- | --- |
| BBBP (SMD) | 23.9 | 1 | SELU-MPNN | 641 MiB | 641 MiB | 641 MiB |
|  |  |  | AMPNN | 673 MiB | 677 MiB | 677 MiB |
|  |  |  | EMNN | 843 MiB | 935 MiB | 935 MiB |
| SIDER (SMD) | 32.3 | 27 | SELU-MPNN | 1825 MiB | 2345 MiB | 2345 MiB |
|  |  |  | AMPNN | 1945 MiB | 2187 MiB | 2187 MiB |
|  |  |  | EMNN | 2361 MiB | 2643 MiB | 2365 MiB |
| Tox21 (SMD) | 19.2 | 12 | SELU-MPNN | 625 MiB | 649 MiB | 649 MiB |
|  |  |  | AMPNN | 649 MiB | 713 MiB | 713 MiB |
|  |  |  | EMNN | 803 MiB | 905 MiB | 903 MiB |

## Example Validation/Test Curves

Below are some example validation and test learning curves, to demonstrate the architecture does not demonstrate overfitting tendencies on the training and test sets. This is a plot of the test set score corresponding to the best-so-far observed validation score, for the split on which the hyperparameter optimisation was run.


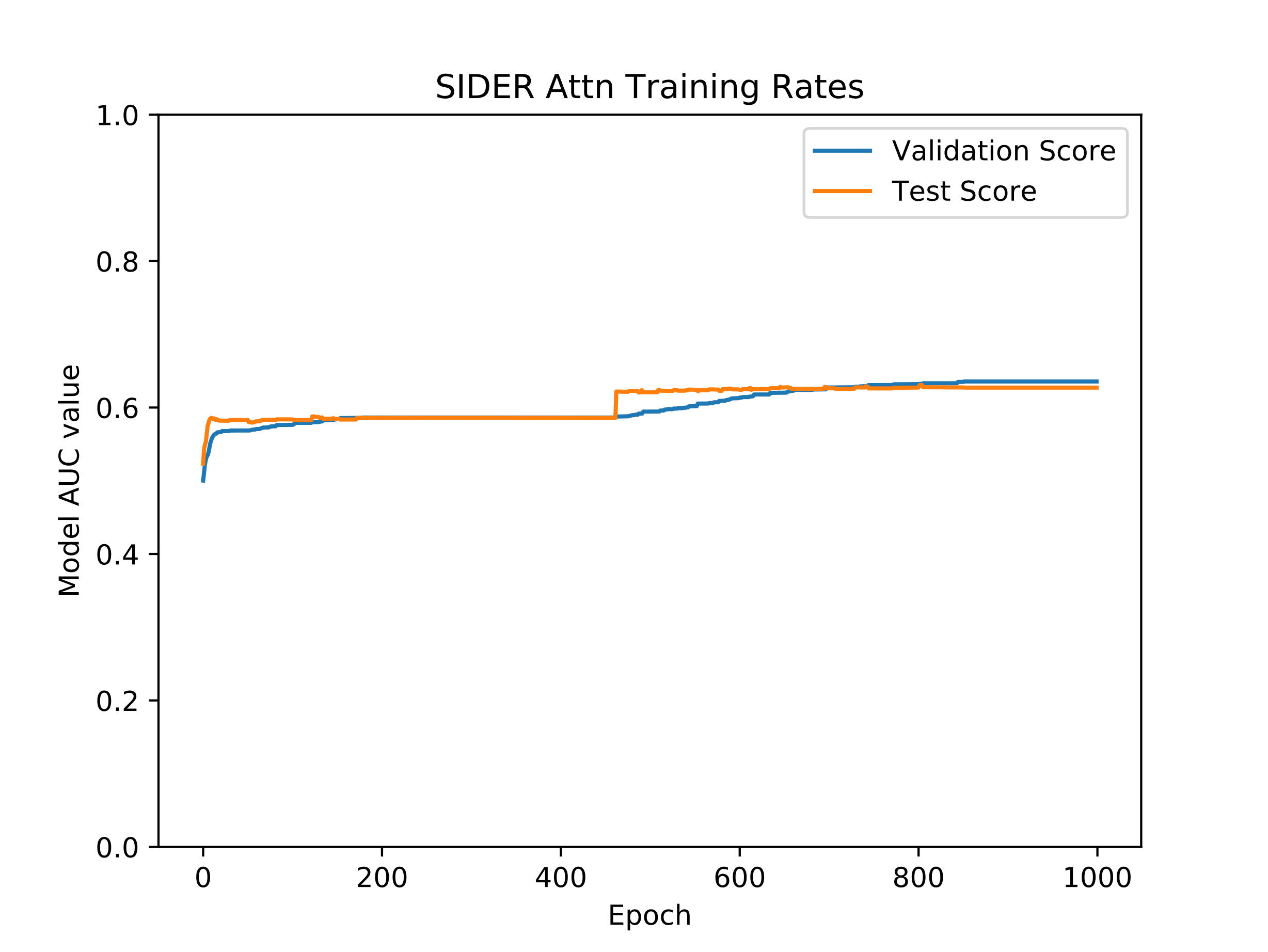

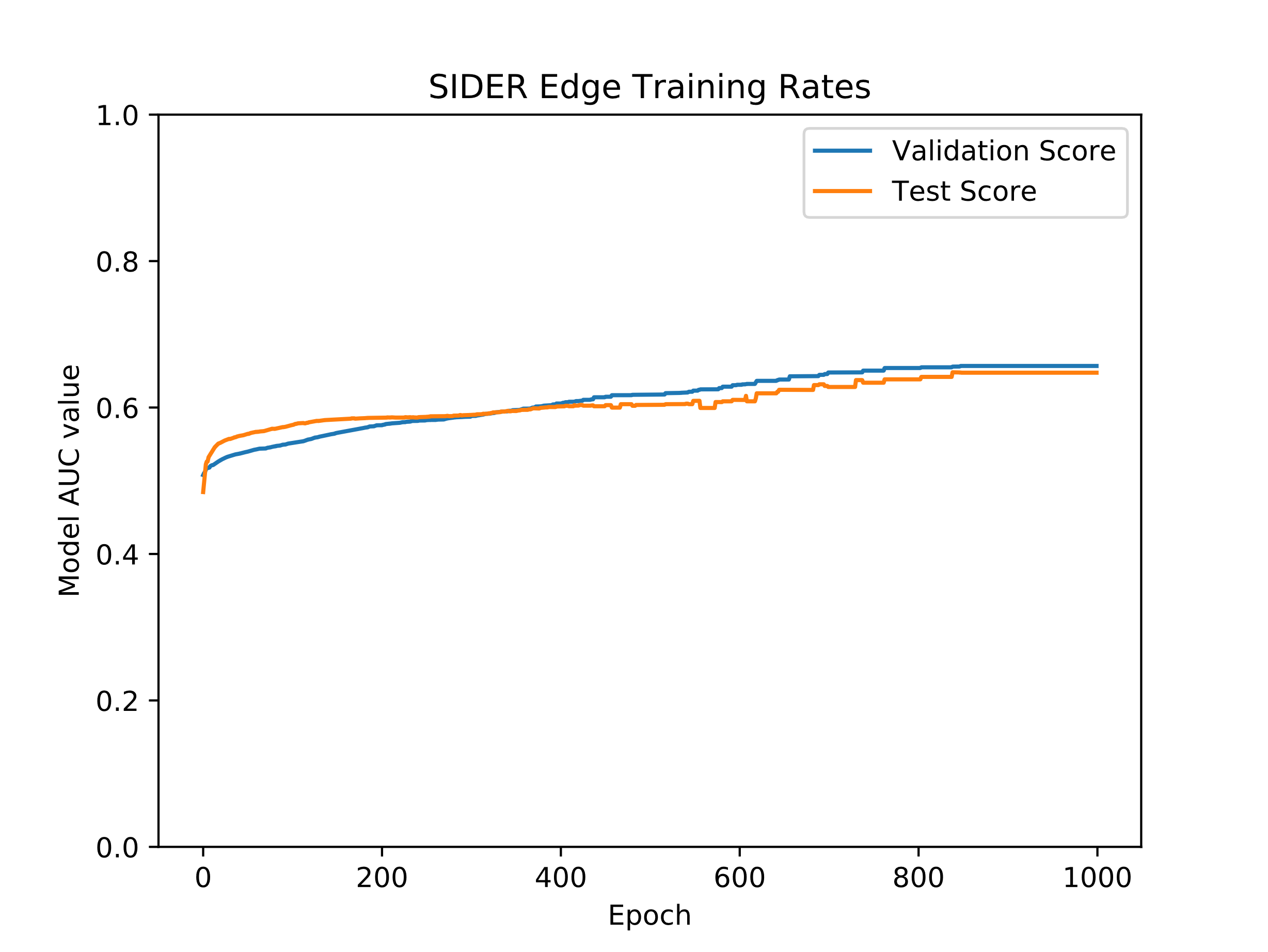


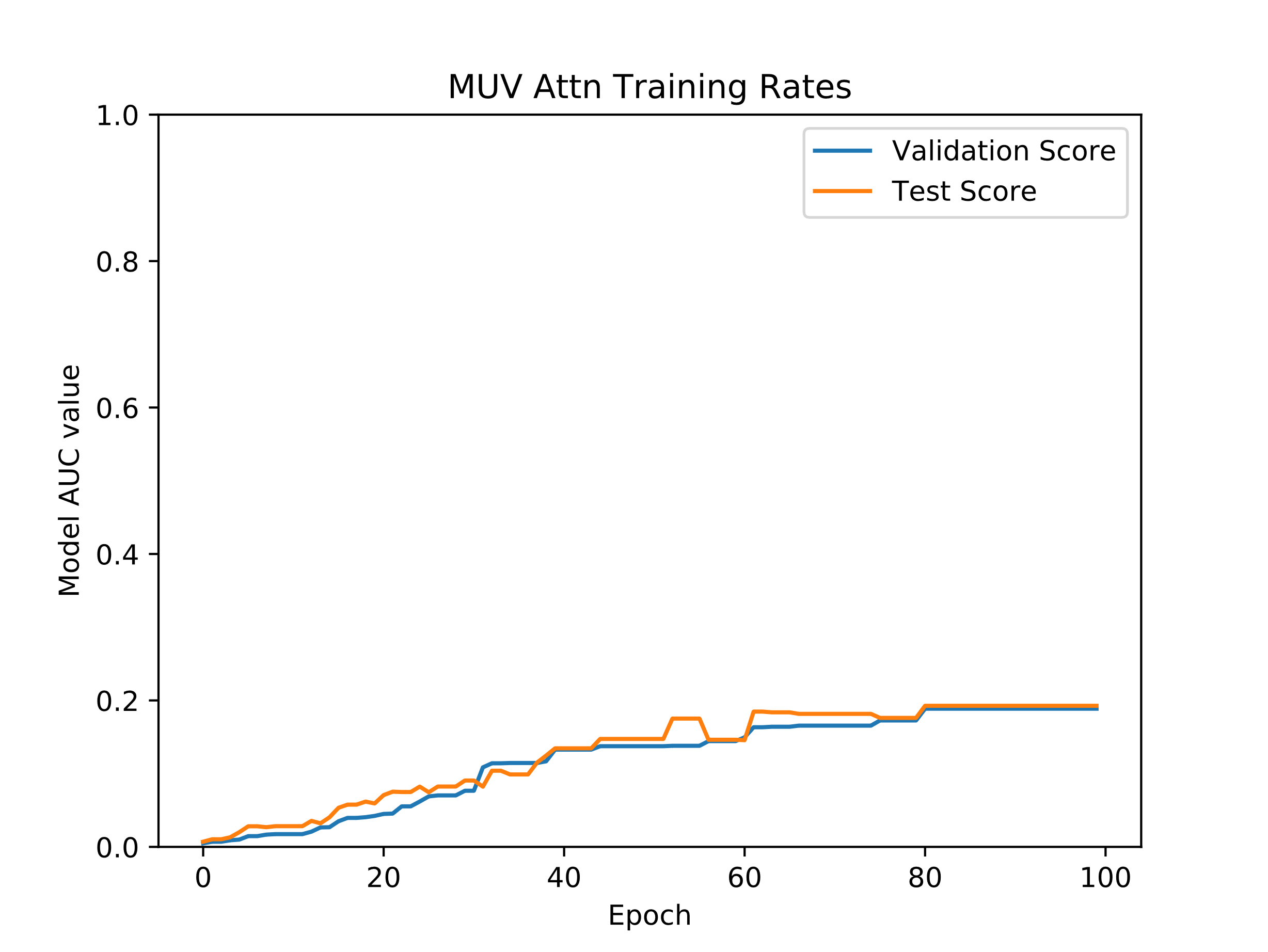

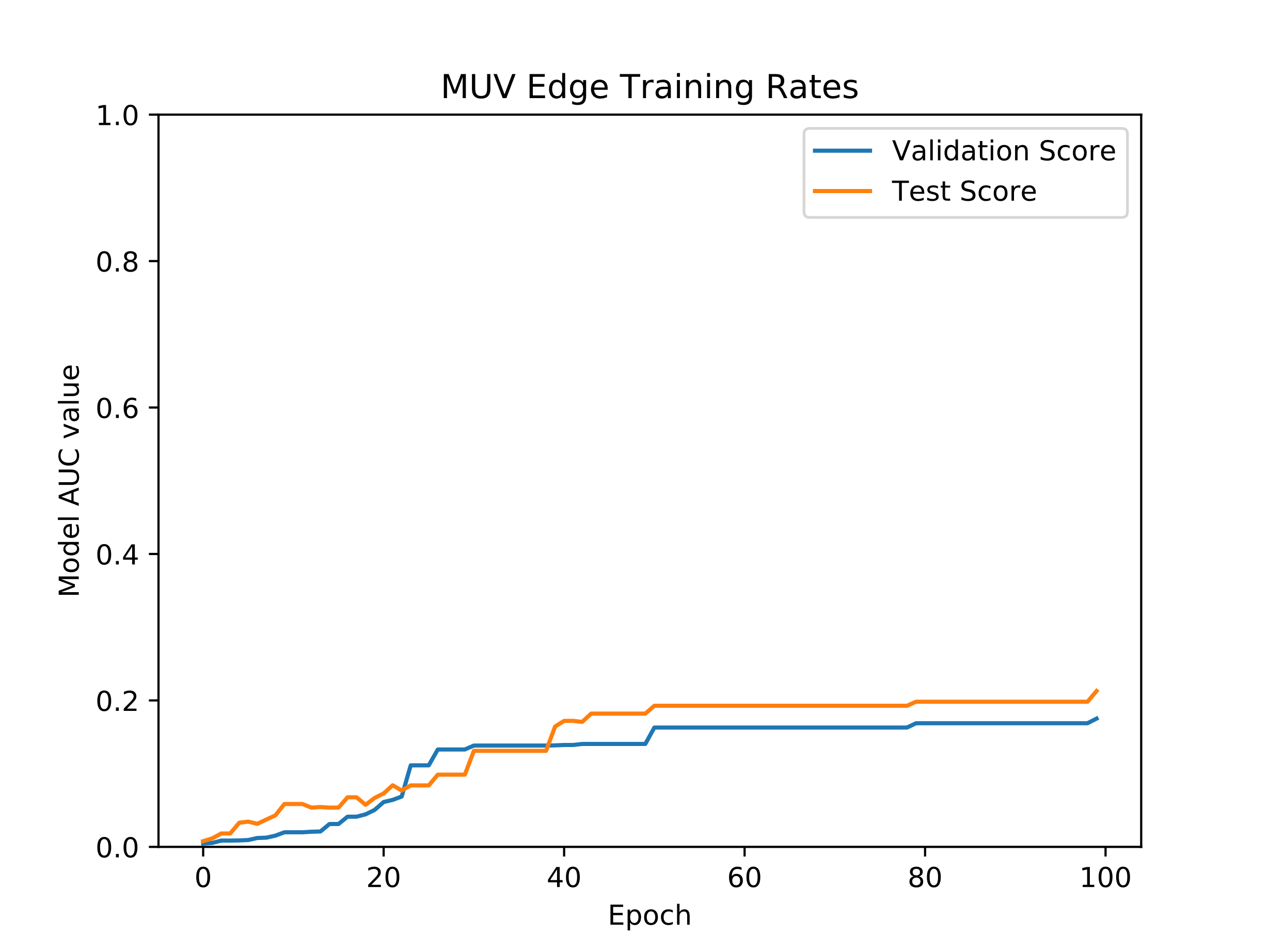


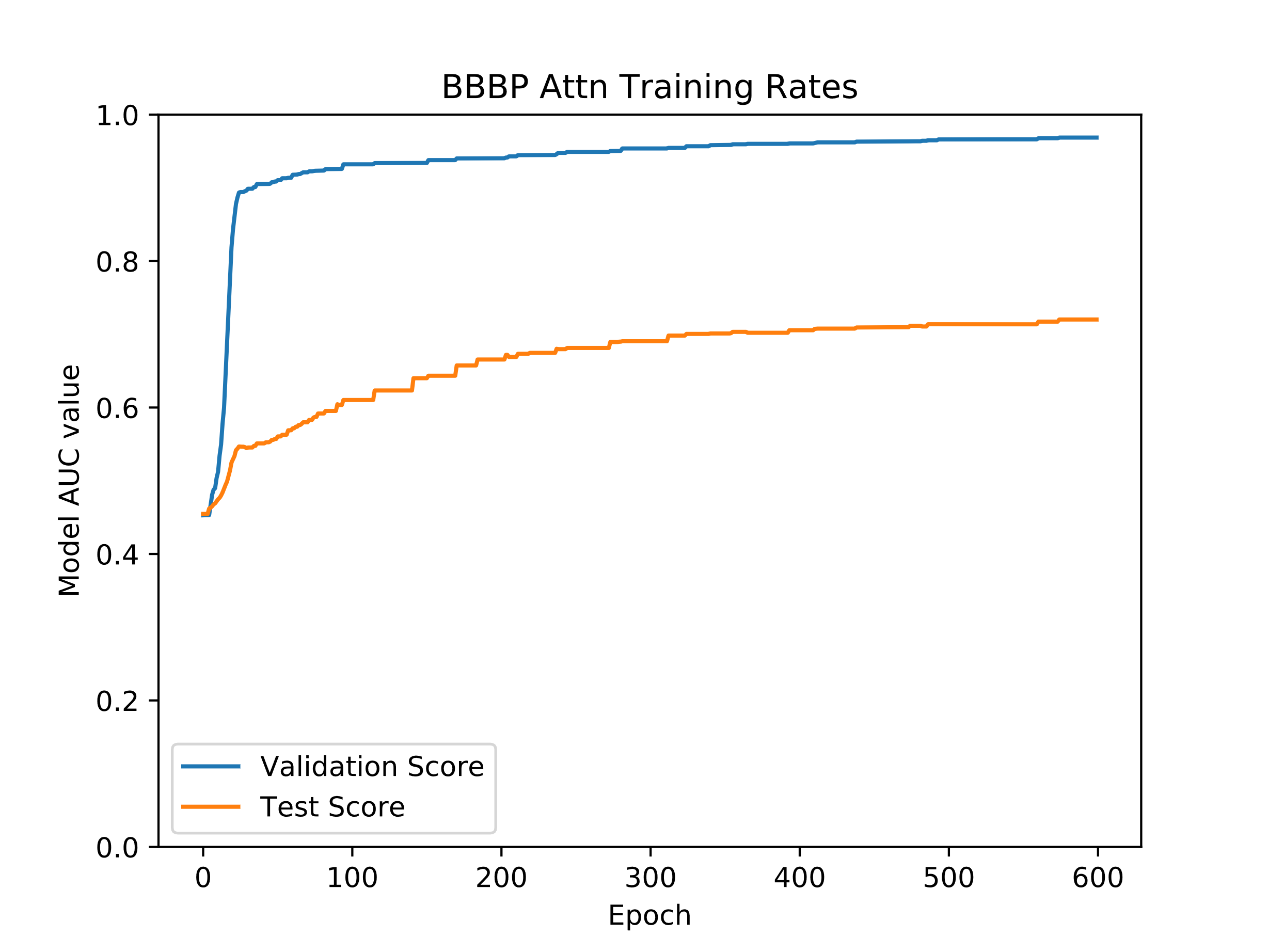

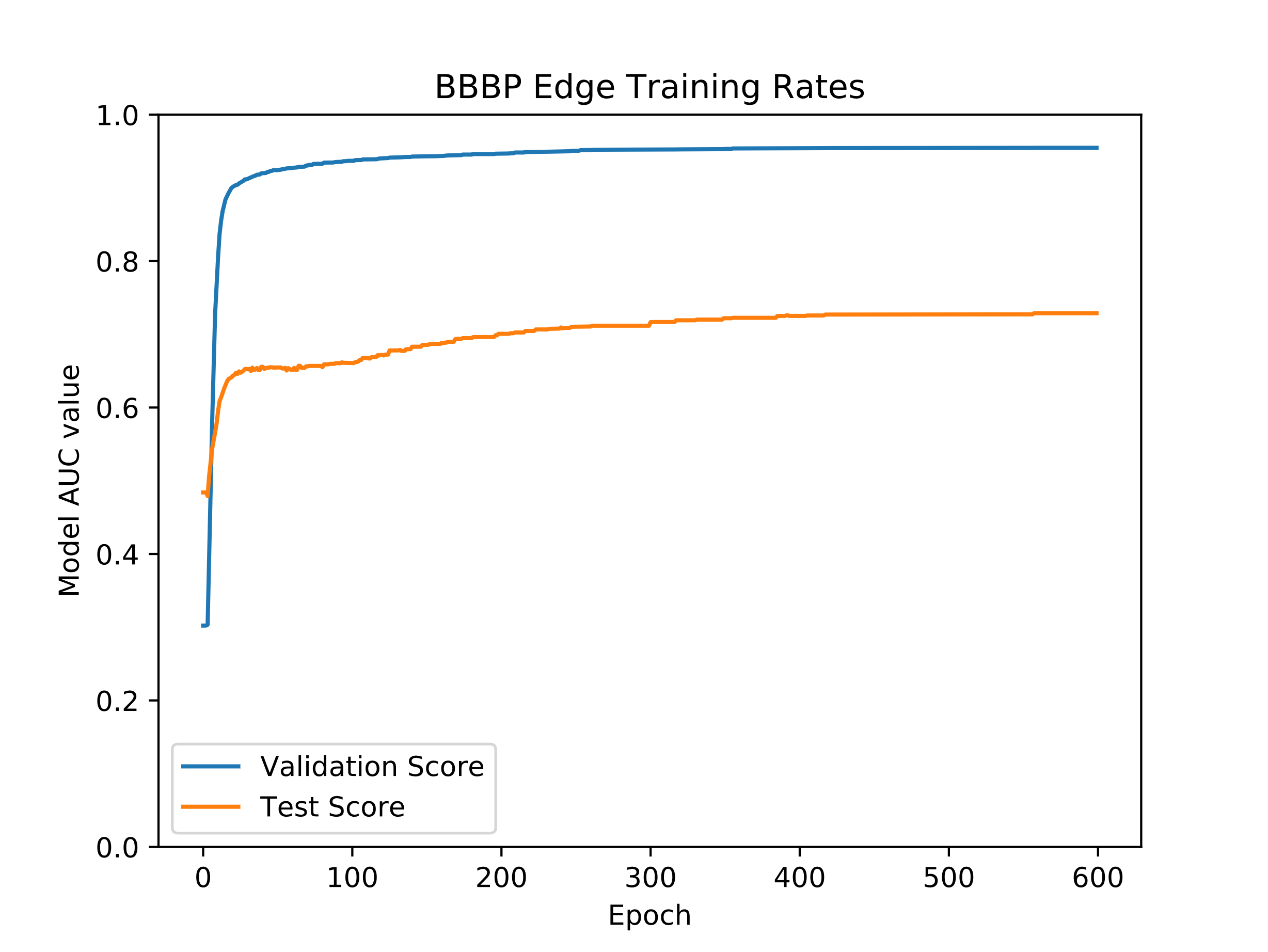


## Dataset Class Imbalances

Here we present additional information regarding dataset class imbalances for both SMD and Original datasets, as referenced in the primary manuscript.


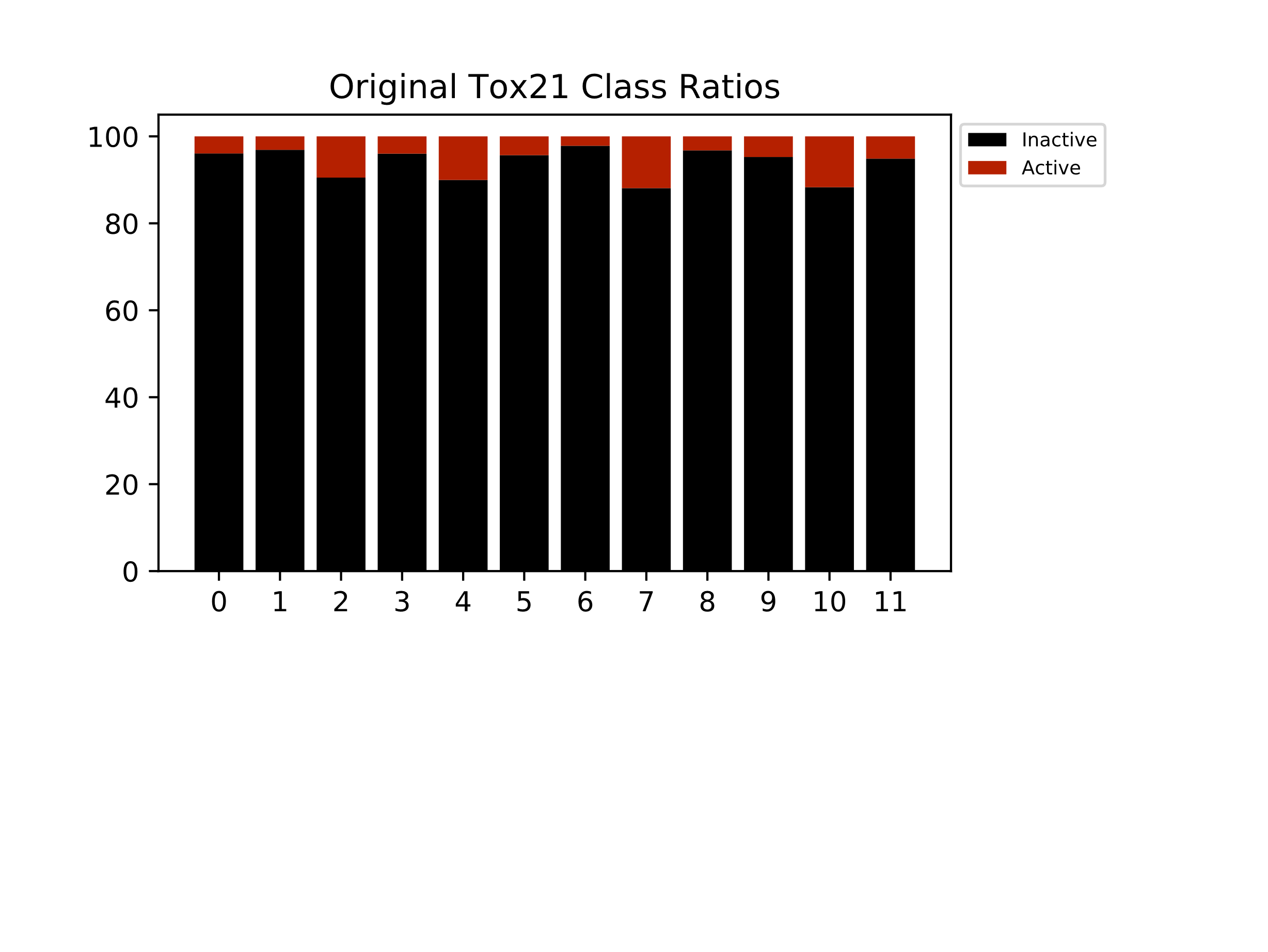

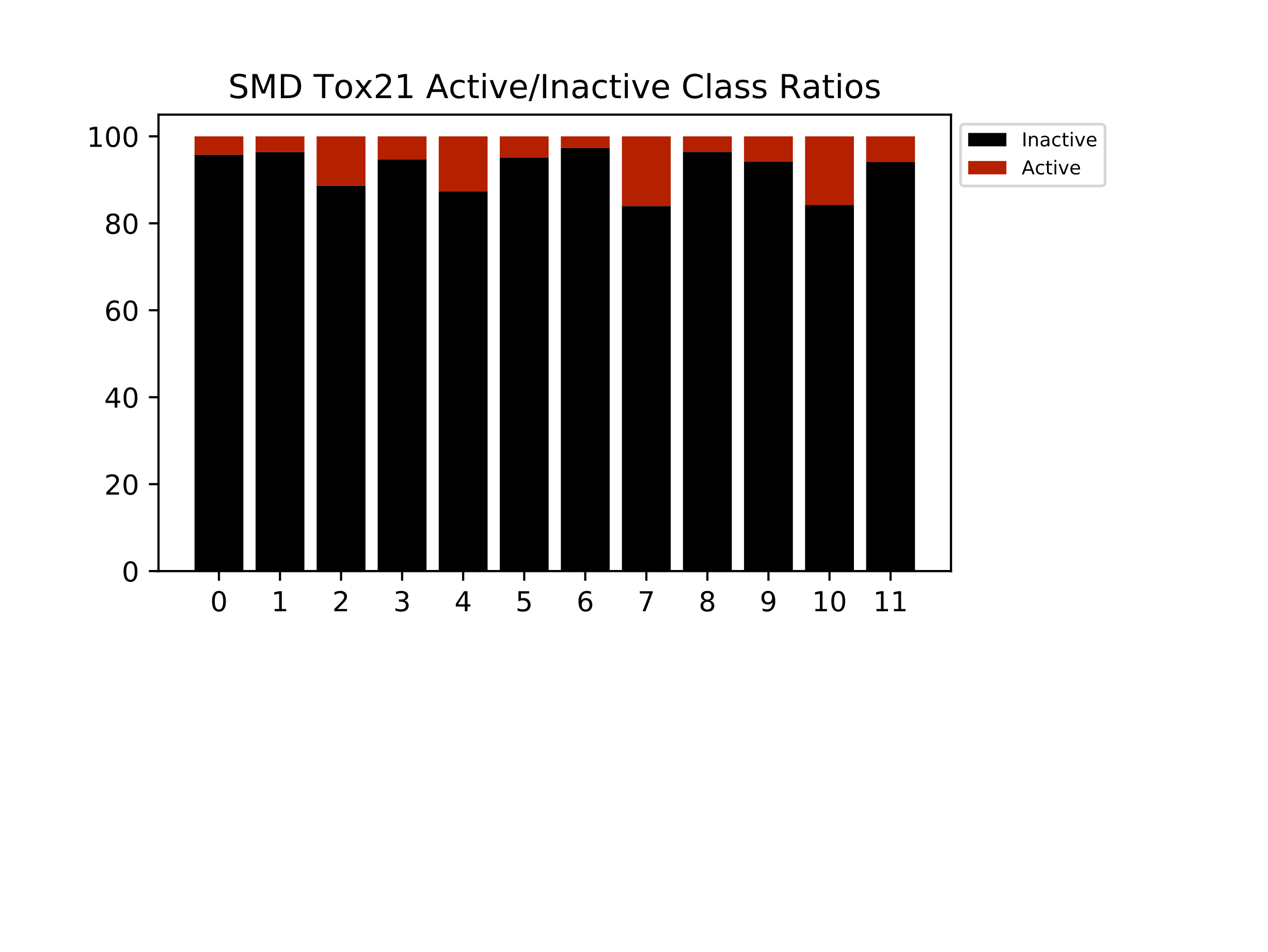

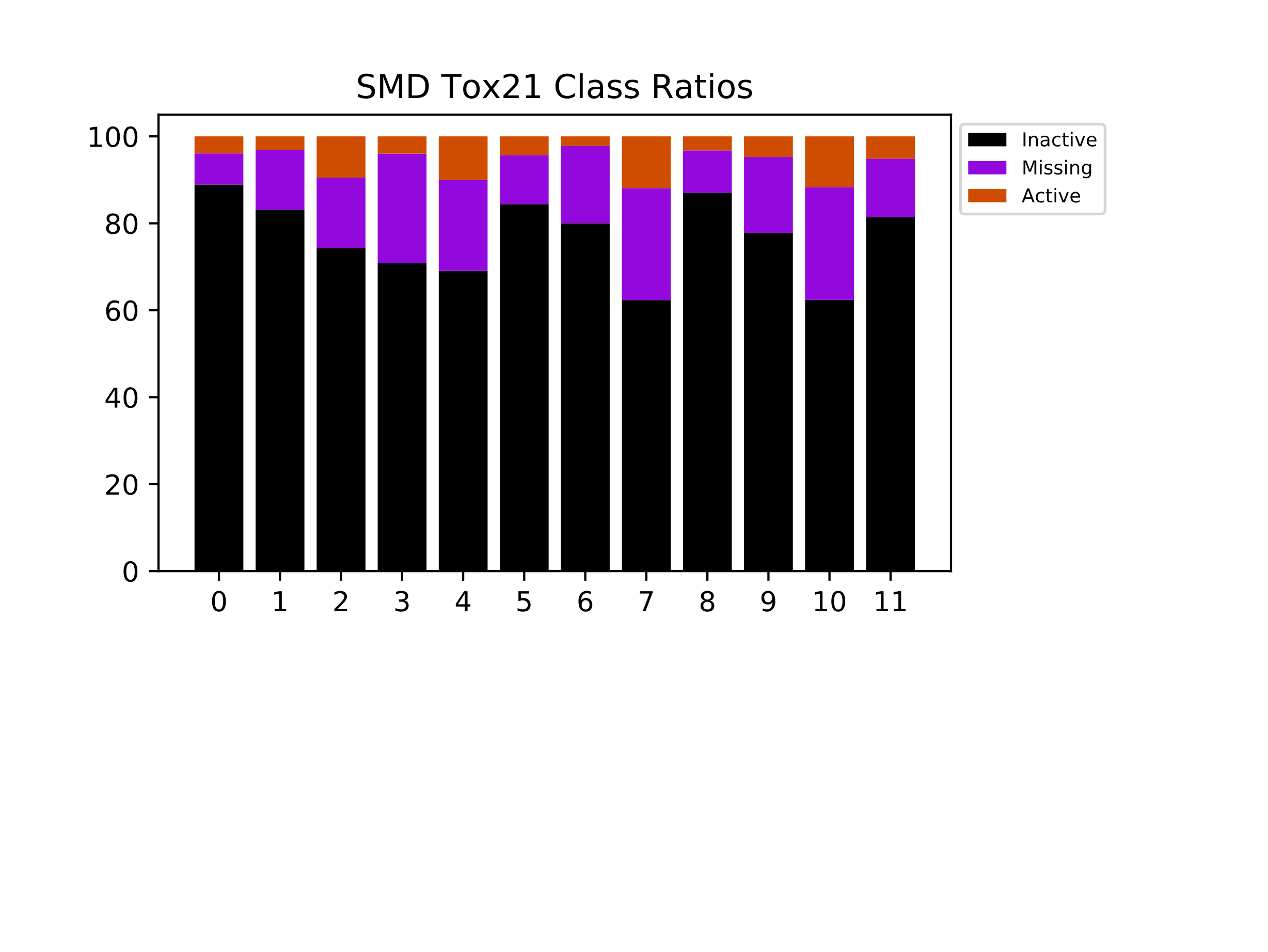


Figure S1 – Tox21 class ratios before and after processing for both preprocessing approaches. The plot on the top shows the ratio of actives and inactives when missing data is imputed as inactive. The plots on the bottom show the SMD ratios – both including missing data (right) and ignoring missing data (left).


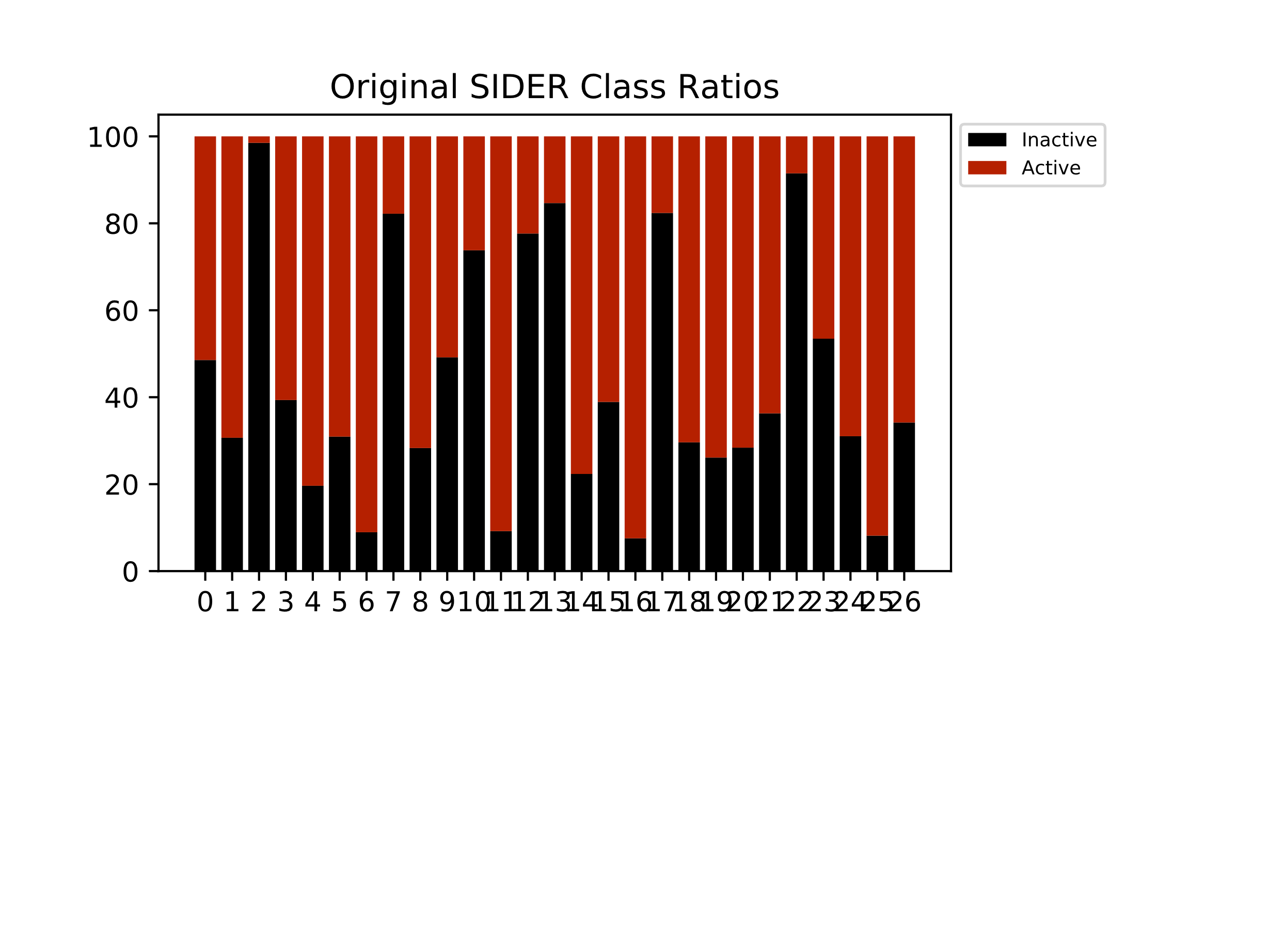


Figure S2 – Ratio of actives and inactives found in each task of the SIDER dataset.
